# Essential Regulation of YAP1 in Fate Determinations of Spermatogonial Stem Cells and Male Fertility by Interacting with RAD21 and Targeting NEDD4 in Humans and Mice

**DOI:** 10.34133/research.0544

**Published:** 2024-12-10

**Authors:** Chunyun Li, Wei Chen, Yinghong Cui, Dong Zhang, Qingqing Yuan, Xing Yu, Zuping He

**Affiliations:** ^1^ Key Laboratory of Model Animals and Stem Cell Biology in Hunan Province, Hunan Normal University School of Medicine; Engineering Research Center of Reproduction and Translational Medicine of Hunan Province; Manufacture-Based Learning and Research Demonstration Center for Human Reproductive Health New Technology of Hunan Normal University, Changsha 410013, China.; ^2^ Shanghai Key Laboratory for Assisted Reproduction and Reproductive Genetics, Center for Reproductive Medicine, Renji Hospital, School of Medicine, Shanghai Jiao Tong University, Shanghai 200135, China.

## Abstract

Spermatogenesis is a sophisticated biological process by which spermatogonial stem cells (SSCs) undergo self-renewal and differentiation into spermatozoa. Molecular mechanisms underlying fate determinations of human SSCs by key genes and signaling pathways remain elusive. Here, we report for the first time that Yes1-associated transcriptional regulator (YAP1) is required for fate determinations of SSCs and male fertility by interacting with RAD21 and targeting NEDD4 in humans and mice. YAP1 was mainly located at cell nuclei of human SSCs. YAP1 silencing resulted in the decreases in proliferation and DNA synthesis as well as an enhancement in apoptosis of human SSCs both in vivo and in vitro. RNA sequencing and real-time polymerase chain reaction assays identified NEDD4 as a target of YAP1, and NEDD4 knockdown inhibited the proliferation of human SSCs and increased their apoptosis. Furthermore, YAP1 interacted with RAD21 to regulate NEDD4 transcription in human SSCs. Importantly, YAP1 abnormalities were found to be associated with non-obstructive azoospermia (NOA) as manifested as lower expression level of YAP1 in testicular tissues of NOA patients and *YAP1* single-nucleotide variants (SNVs) in 777 NOA patients. Finally, *Yap1* germline conditional knockout (cKO) mice assumed mitotic arrest, low sperm count, and motility. Collectively, these results highlight a critical role of YAP1 in determining the fate determinations of human SSCs and male infertility through the YAP1/RAD21/NEDD4 pathway. This study provides new insights into the genetic regulatory mechanisms underlying human spermatogenesis and the pathogenesis of NOA, and it offers new targets for gene therapy of male infertility.

## Introduction

Infertility is a prevalent disease affecting 8% to 12% of couples around the world [1], and notably, it causes severe social, psychosocial, and economic burdens. Male factor accounts for around half of these cases, and the incidence of infertility has been increased by 0.29% annually [2]. Non-obstructive azoospermia (NOA), the most severe condition of male infertility, represents approximately 60% of azoospermia cases and constitutes 10% of infertile men [3], and aberrant spermatogenesis is the most common cause of NOA [1].

Spermatogenesis is a sophisticated biological process by which spermatogonial stem cells (SSCs) undergo self-renewal and differentiate into mature spermatozoa. Genetics and epigenetic factors regulate the fate determinations of SSCs, including cellular self-renewal, differentiation, and apoptosis [4,5]. Currently, there are more than 1,000 genes related to male infertility in the mouse genome informatics database of Jackson Laboratory, and 435 genes of them are involved in fate determinations of mouse SSCs [6], e.g., *ID4* [7], *Sall4* [8], *Mast4* [9], *Nanos2* [10], *Six6os1* [11], *Cfap47* [12], and *Serbp1* [13]. However, in humans, only a small number of genes have been implicated in male infertility [14], including *DNAH10* [15], *MSH5* [16], and *TDRD7* [17]. Compared to mouse SSCs, it remains challenging to better understand the molecular mechanisms governing human SSC fate determinations. This is due to the limited availability of human testicular tissues for research and notable differences in cell types and characteristics between human and rodent SSCs. We have recently shown that OIP5 [18], PAK1 [19], RNF144B [20], USP11 [21], and KLF2 [22] control the proliferation and apoptosis of human SSCs. However, the molecular mechanisms by which key genes regulate the fate determinations of human SSCs and the pathogenesis of NOA remain largely elusive.

We have previously shown that the PAK1/PDK1 pathway regulates the proliferation and apoptosis of human SSCs [19]. Interestingly, we observed that YAP1 (Yes1-associated transcriptional regulator) could be decreased by PDK1 knockdown in the human SSCs (Fig. S1A to E), implicating that YAP1 may play a role in the fate determinations of human SSCs. YAP1, first discovered in *Drosophila*, serves as an important downstream effector of the Hippo signaling pathway. This pathway regulates organ size and tissue homeostasis by modulating stemness, cell proliferation, and apoptosis [23]. Due to lacking of DNA-binding domain, YAP1 frequently functions as a coactivator of transcription. Cascade kinases from the Hippo pathway, including SAV1, MST1/2, MOB1, and LATS1/2, mediate the activity of YAP1 as inhibitors [23,24]. Interestingly, the nucleocytoplasmic shuttling of YAP1 is critical for its biological activity [25]. When Hippo signaling is blocked, YAP1 trans-localizes into cell nuclei and binds to transcriptional factors, e.g., TEAD, Smad, and p63/p73, which controls the expression of its targeting genes. Unlike most of other proteins, YAP1 undergoes phosphorylation and binds to the 14-3-3 proteins in cell cytoplasm, which subsequently triggers the ubiquitination and degradation of YAP1. It has been indicated that the Hippo pathway is necessary for ovarian granulosa cell fate decision [26,27]. Male infertility has been observed in mice with both Mst1 and Mst2 deficiency in the epididymal epithelium [28]. Meanwhile, YAP1 participates in the fate determinations of various stem cells [29]. However, the functions and molecular mechanisms of YAP1 in mediating human SSC fate determinations await to be elucidated.

In this study, we are the first to report that the expression level of YAP1 was lower in NOA testicular tissues compared to obstructive azoospermia (OA) patients with normal spermatogenesis, and notably, there was relevance between single-nucleotide variants (SNVs) of *YAP1* and the risk of NOA. Notably, we demonstrated that *YAP1* mutation (c.680C>T, S227L) led to the protein instability of YAP1, the decrease in proliferation, and an increase in apoptosis of human SSCs. We next revealed that YAP1 silencing leads to the decrease in the proliferation and an enhancement in apoptosis of human SSCs both in vivo and in vitro. Furthermore, we found that YAP1 could bind to RAD21 to regulate the downstream gene *NEDD4*. Additionally, *Yap1* conditional knockout mice in the germline (*Yap1* cKO) exhibited mitosis arrest, the decreases in mature sperm count and motility, abnormal sperm morphology, and an increase in apoptosis of male germ cells. Collectively, this study is of unusual significance, because it provides a novel genetic regulatory mechanism and molecular network underlying human SSC fate determinations and the pathology of azoospermia and it offers new biomarkers for gene targeting and diagnosis of male infertility.

## Results

### YAP1 is expressed at a lower level in the testes of NOA patients than normal men, and SNVs of *YAP1* are associated with NOA

**Fig. 1.**
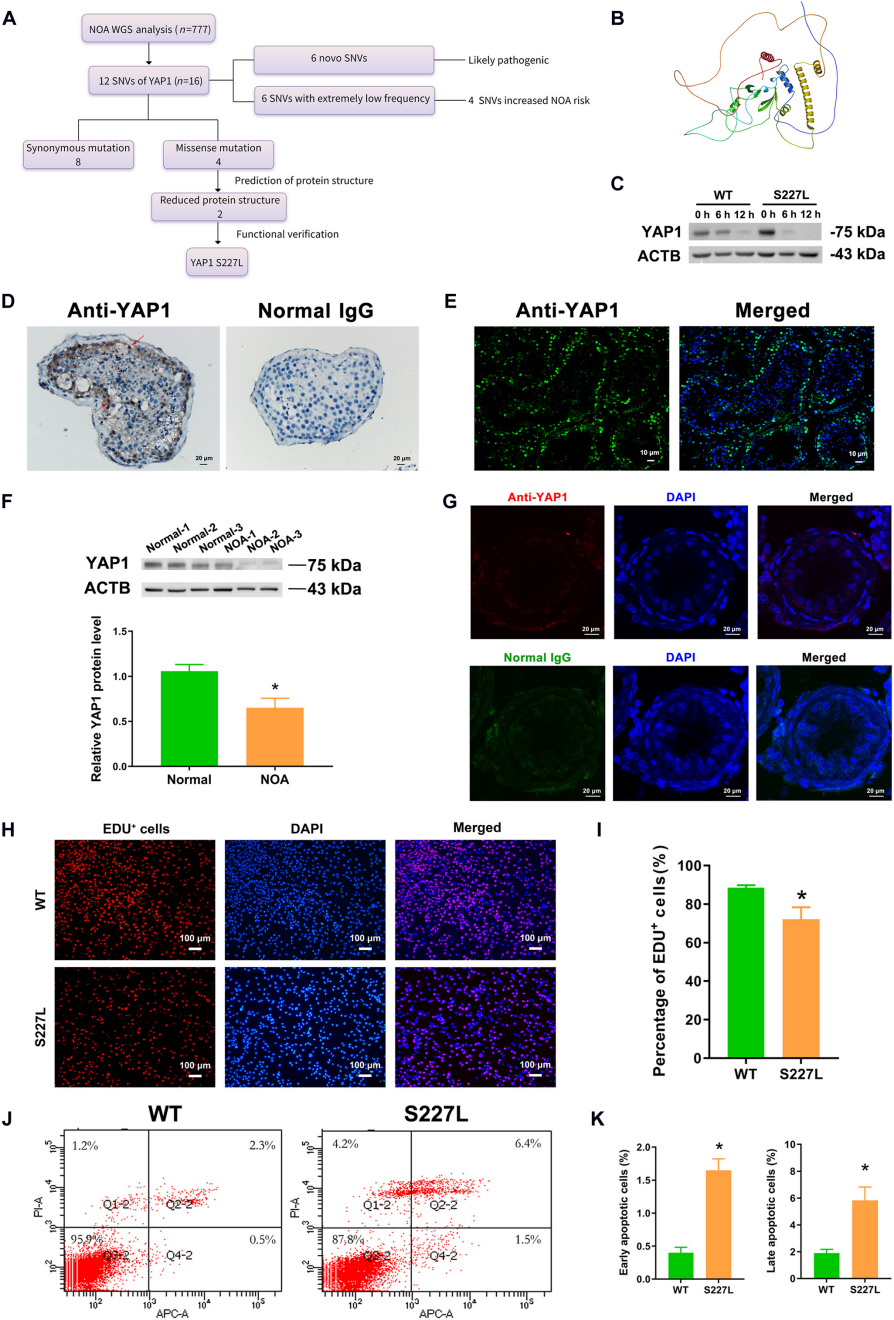
The expression of YAP1 in the testes of OA patients with normal spermatogenesis and NOA patients and the role of YAP1 S227L mutation in regulating the proliferation and apoptosis of human SSCs. (A) Schematic diagram of WES in 777 NOA patients. (B) Influence of *YAP1* mutation p.S227L on the structure of YAP1 protein. (C) Protein stability experiment of WT and *YAP1* S227L mutation. (D and E) Immunofluorescence (D) and immunohistochemical staining (E) showed cellular localization of YAP1 in human normal seminiferous tubules. Normal IgG antibody replacing YAP1 was utilized as a negative control. Scale bars in (D), 20 μm. (F) Western blots displayed the expression levels of YAP1 in the testes from the OA patients with normal spermatogenesis and NOA patients. *P < 0.05. (G) Immunofluorescence indicated the expression and localization of YAP1 in NOA testis tissues. Scale bars, 20 μm. (H and I) EDU incorporation assay displayed DNA replication of human SSCs with the S227L mutation of *YAP1* and WT of *YAP1*. Scale bars, 100 μm. (J and K) Annexin V–APC/PI and flow cytometry analysis demonstrated the apoptosis of human SSCs treated with S227L mutation and WT of *YAP1*.

To investigate the biological relevance of YAP1 and NOA, whole-exome sequencing (WES) was performed with 777 patients diagnosed with NOA (Fig. 1A). Interestingly, we found that 16 patients (16/777) had *YAP1* (NM_001282101.2) SNVs among 777 NOA patients, and this incidence of *YAP1* by 2.06% was remarkably higher when compared to the average gene mutation rate by 0.3%. The minor allele frequencies (MAFs) of all the variants were lower than 0.01 in the Genome Aggregation Database (gnomAD), 1000 Genomes Project (1000G), and ExAC. Notably, among these 16 patients, there were 12 SNVs, including 6 novel SNVs with absent (*YAP1* c.42G>A, *YAP1* c.55C>A, *YAP1* c.134C>T, *YAP1* c.1116C>G, *YAP1* c.1482G>A, and *YAP1* c.689-10T>C) and 6 SNVs with extremely low frequency (*YAP1* c.57G>A, *YAP1* c.387A>G, *YAP1* c.567C>T, *YAP1* c.680C>T, *YAP1* c.879C>T, and *YAP1* c.1016C>T) in the above databases. Among all SNVs, there was one homozygous variant *YAP1* (*YAP1* c.55C>A) and the remaining variants were heterozygous (Table S1). Next, we conducted the correlation analyses between the SNV allele frequency of *YAP1* gene and the risk of NOA. According to the American College of Medical Genetics and Genomics (ACMG) guidelines, 6 variants were categorized as likely pathogenic (PM2). For the other 6 SNVs with extremely low frequency, statistical analyses were performed. The allele frequency of the same locus in the GnomAD database was used as the control, and odds ratios (ORs) and the confidence interval (CI) around the OR were chosen as the indicators for risk assessment. Statistical analysis demonstrated that the carriage of G allele at the rs773991529 locus (OR = 40.445, 95% CI = 4.518 to 362.059), T allele at the rs757399180 locus (OR = 23.059, 95% CI = 2.835 to 187.526), T allele at the rs756175059 locus (OR = 26.926, 95% CI = 3.240 to 223.780), and T allele at the rs527326391 locus (OR = 9.685, 95% CI = 1.007 to 93.171) was found to be associated with increasing risk of NOA (PS4), while the carriage of A allele at the rs937455182 locus might decrease the risk of NOA (OR = 0.025, 95% CI = 0.003 to 0.174). There was no correlation between the carriage of T at the rs376161041 locus and NOA risk (*P* > 0.05). Subsequently, the pathogenicity of missense variants of *YAP1* was predicted and evaluated using Sorting Intolerant from Tolerant (SIFT), PolyPhen-2_HDIV, MutationTaster, and M-Cap. As shown in Table S2, c.55C>A, c.134C>T, c.680C>T, and c.1016C>T in the *YAP1* gene were potentially deleterious variants (PP3). In addition, analysis of impact of single-nucleotide polymorphisms on protein stability predicted from the protein structure (AF-P46937-F1, AlphaFold) was conducted using the INPS-MD server (Biocomputing Group) and the MAESTROweb server. The S227L mutant appeared to remarkably impair protein stability, with an increase in stability change (DDGpred) in 1.5 and 0.4 kcal/mol, respectively (Fig. 1B). To verify the prediction results, we constructed P45L and S227L mutations of *YAP1* (Fig. S2A) in a human SSC line [19]. The human SSC line displayed phenotypic characteristics similar to human primary SSCs (Fig. S3A and B). The protein stability experiment showed that the stability of YAP1 was decreased after *YAP1* S227L mutations treated with cycloheximide (CHX) in human SSCs (Fig. 1C).

We determined the cellular distribution of YAP1 in normal human testicular tissues. Immunohistochemistry via 3,3′-diaminobenzidine (DAB) staining indicated that YAP1 was predominantly localized in cell nuclei of human primary SSCs (arrows) around the basement membrane of seminiferous tubules in the normal testicular tissues (Fig. 1D, left panel). A few of pachytene spermatocytes and round spermatids were positive for YAP1 (Fig. 1D, left panel). Immunohistochemistry via immunofluorescence further demonstrated that YAP1 was predominantly located in the nuclei of primary SSCs (Fig. 1E). We compared YAP1 expression levels in testes from NOA patients and OA patients with normal spermatogenesis. Western blots showed a lower level of YAP1 protein in testicular tissues from NOA patients compared to OA patients with normal spermatogenesis (Fig. 1F). Immunohistochemistry displayed that YAP1 immunostaining could not be seen in NOA patients (Fig. 1G), which was distinct from YAP1 immunostaining in OA patients with normal spermatogenesis (Fig. 1E).

Whether P45L and S227L mutations of *YAP1* affect DNA synthesis and apoptosis of human SSC, we performed 5-ethynyl-2′-deoxyuridine (EDU) incorporation assays and Annexin V/propidium iodide (PI) staining via flow cytometry. EDU incorporation assays showed that S227L mutation of *YAP1* decreased DNA synthesis of human SSCs (Fig. 1H and I), and Annexin V/PI staining via flow cytometry indicated the increased apoptosis in the human SSCs compared to wild type (WT) of *YAP1* (Fig. 1J and K). However, P45L mutation of *YAP1* had no effect on DNA synthesis and apoptosis of human SSCs (Fig. S2B and E). Collectively, these results implicate that *YAP1* SNVs and/or lower expression level are associated with the NOA and that *YAP1* mutations affect the fate determinations of human SSCs.

### YAP1 knockdown inhibits proliferation and DNA synthesis of human SSCs, enhances their apoptosis in vitro, and suppresses self-renewal of human SSCs in vivo

**Fig. 2.**
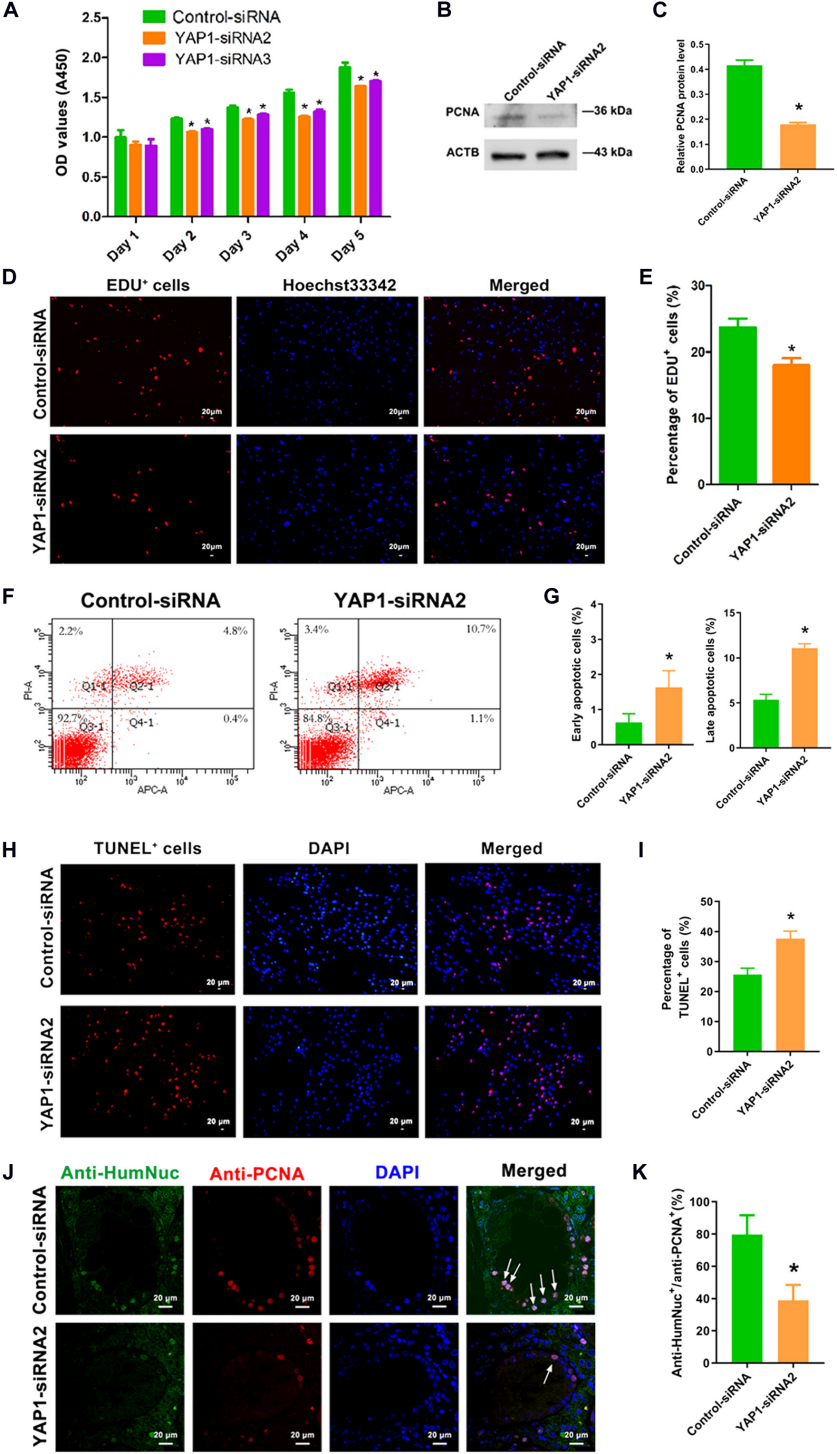
The effect of YAP1 silencing on controlling the proliferation and apoptosis of human SSCs. (A) CCK-8 assay indicated the growth activity of human SSCs after treatment of YAP1-siRNA2 and YAP1-siRNA3. (B and C) Western blots demonstrated the expression of PCNA protein in human SSCs after YAP1-siRNA2 and control-siRNA transfection. (D and E) EDU incorporation assay displayed the EDU-positive cells in human SSCs affected by YAP1-siRNA2 and control-siRNA. Cell nuclei were stained with Hoechst 33342. Scale bars, 20 μm. (F and G) Annexin V–APC/PI and flow cytometry analysis revealed the apoptosis in human SSCs affected by YAP1-siRNA2 and control-siRNA for 48h. (H and I) TUNEL staining showed that the number of TUNEL-positive cells in human SSCs transfected by YAP1-siRNA2 and control-siRNA. Scale bars, 20 μm. (J and K) Double immunostaining illustrated coexpression of HumNuc and PCNA in seminiferous tubules of the recipient mice transplanted with human SSCs with control-siRNA and YAP1-siRNA2 transfection. Scale bars, 20 μm. *P < 0.05.

Subsequently, we investigated the impact of YAP1 silencing on human SSC proliferation and apoptosis using RNA interference (RNAi). Three small interfering RNAs (siRNAs) were designed to target human *YAP1* mRNA, and their detailed information was listed in Table S5. Cy3-labeled siRNA was employed to illustrate that the transfection efficiency of siRNAs in human SSCs was more than 90% (Fig. S4A). Compared to control-siRNA, YAP1-siRNA2 had a higher knockdown efficiency in human SSCs via real-time polymerase chain reaction (PCR) (Fig. S4B) and Western blots (Fig. S4C and D). As such, YAP1-siRNA2 was chosen for further studies of YAP1 functions and mechanisms. Our cell counting kit-8 (CCK-8) assay showed a decrease in the proliferation potential of human SSCs at 48 to 120 h after transfection with YAP1-siRNA2 and YAP1-siRNA3 (Fig. 2A). Western blots indicated a reduction in the level of proliferating cell nuclear antigen (PCNA) in human SSCs following treatment with YAP1-siRNA2 (Fig. 2B and C). In EDU incorporation assays, the percentages of EDU-positive cells were lower in human SSCs treated with YAP1-siRNA2 than in control-siRNA (Fig. 2D and E). Annexin V/PI staining with flow cytometry revealed that YAP1-siRNA2 remarkably elevated both early and late apoptosis of human SSCs (Fig. 2F and G). Moreover, the proportion of terminal deoxynucleotidyl transferase–mediated deoxyuridine triphosphate nick end labeling (TUNEL)-positive cells was higher in human SSCs with YAP1-siRNA2 treatment than with control-siRNA (Fig. 2H and I). Considered together, these findings imply that YAP1 silencing reduces the proliferation and DNA synthesis and enhances the apoptosis of human SSCs.

To examine the role of YAP1 in controlling human SSC fate determinations in vivo, xenotransplantation with human SSCs with control-siRNA and YAP1-siRNA2 was performed. At 4 weeks after treatment of busulfan, testicular volumes were obviously reduced in mice (Fig. S4E). Hematoxylin and eosin (H&E) staining indicated the complete absence of male germ cells within the seminiferous tubules of recipient mice, whereas all types of male germ cells were observed in the normal mice (Fig. S4F). Transplantation of human SSCs with treatment of control-siRNA and YAP1-siRNA2 was conducted through the efferent ducts (Fig. S4G). Immunohistochemistry of testis sections displayed the percentages of human nuclear antigen (HumNuc)-positive cells and PCNA-positive cells were obviously decreased in the recipient mice transplanted with human SSCs treated with YAP1-siRNA2 compared to the control mice (Fig. 2J and K). The percentages of HumNuc- and UCHL1-postive cells exhibited a similar trend by YAP1-siRNA2 (Fig. S4H). Together, these results indicate that YAP1 silencing suppresses the self-renewal of human SSCs in vivo.

### NEDD4 has been identified as a target of YAP1 in human SSCs

**Fig. 3.**
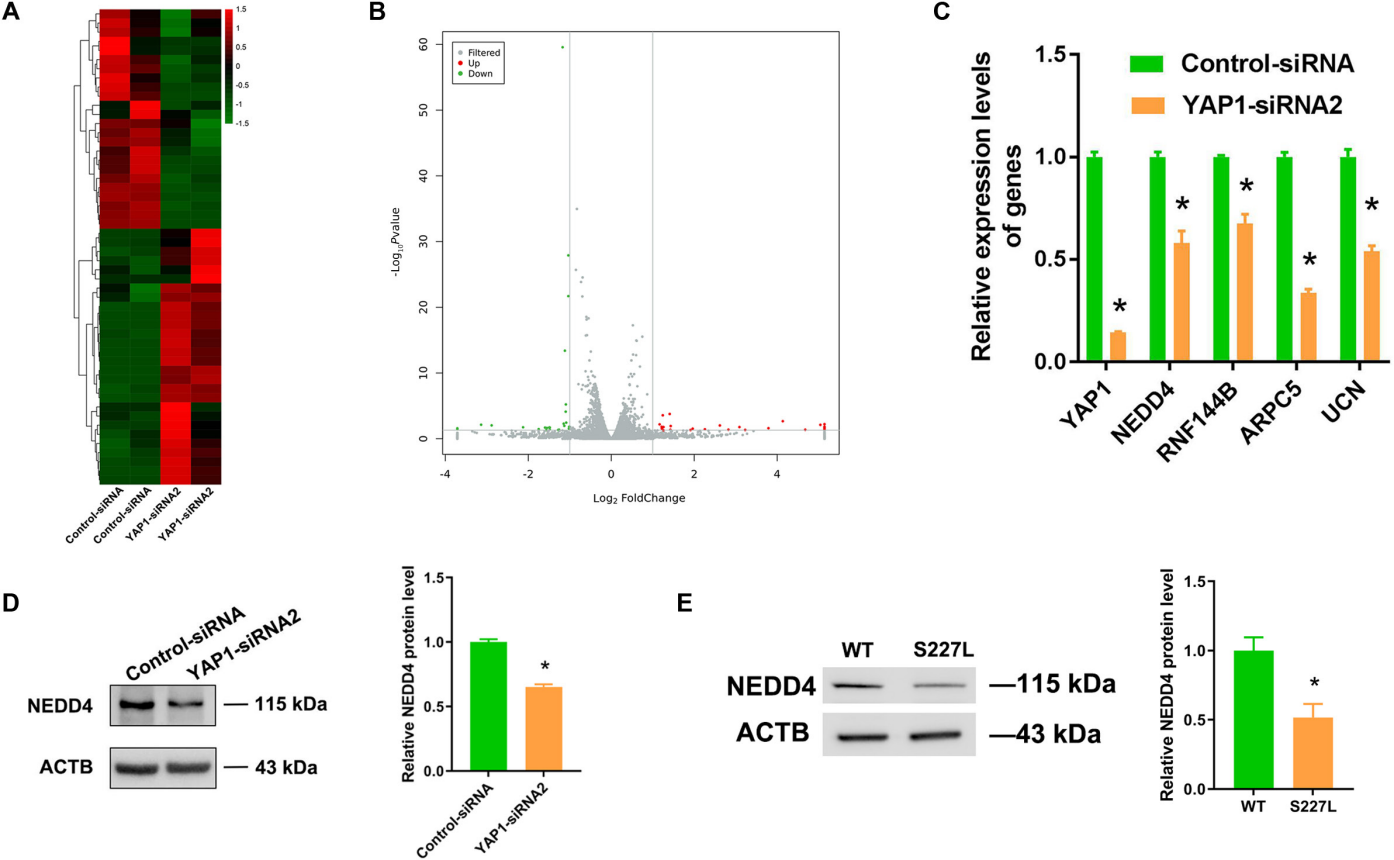
Identification of target genes of YAP1 in human SSCs. (A and B) The clustered heatmap (A) and volcano plot (B) visualized the DEGs in human SSCs between control-siRNA and YAP1-siRNA2. The gray dots denoted the not differentially expressed genes. The red and green dots indicated the up-regulated genes and down-regulated genes, respectively. (C) Real-time PCR displayed the levels of *YAP1*, *NEDD4*, *RNF144B*, *ARPC5*, and *UCN* mRNA in human SSCs transfected with control-siRNA and YAP1-siRNA2. (D) Western blots showed that the expression levels of NEDD4 in human SSC transfection with control-siRNA and YAP1-siRNA2. (E) Western blots showed the expression levels of NEDD4 in WT and S227L of *YAP1* mutation. *P < 0.05.

In order to identify the targets of YAP1 in human SSCs, RNA sequencing was performed to detect the differentially expressed genes (DEGs) of human SSCs between YAP1-siRNA2 and control-siRNA. To ensure RNA quality, we captured electropherograms using the Agilent Bioanalyzer and utilized the RNA integrity number (RIN) to evaluate RNA quality. The electropherograms of human SSCs with treatment of control-siRNA and YAP1-siRNA2 displayed flat baselines and sharp peaks (Fig. S5), and the RIN of 2 groups was 9.5 and 9.6, respectively, which reflects a high integrity of RNA extracted from human SSCs. RNA sequencing revealed that 14,747 and 14,754 genes were present in human SSCs treated with control-siRNA and YAP1-siRNA2, respectively. Based on fold change < 0.5 or fold change > 2, clustered heatmap and volcano plot indicated the DEGs in human SSCs treated with YAP1-siRNA2 and control-siRNA (Fig. 3A and B). According to the criteria [3 < fragments per kilobase of transcript per million mapped reads (FPKM) values < 50, *P* < 0.01, and fold change < 0.5], Gene Ontology (GO) and Kyoto Encyclopedia of Genes and Genomes (KEGG) analyses, *NEDD4*, *RNF144B*, *ARPC5*, and *UCN* were selected as candidate genes for further validation. Real-time PCR showed that mRNA levels of *YAP1*, *NEDD4, RNF144B*, *ARPC5*, and *UCN* were decreased by YAP1-siRNA2 in human SSCs compared to control-siRNA (Fig. 3C), which was in agreement with our findings using RNA sequencing. Moreover, YAP1-siRNA2 remarkably reduced the protein level of NEDD4 compared to control-siRNA (Fig. 3D). Likewise, the S227L mutation of *YAP1* decreased the NEDD4 protein level compared to WT of *YAP1* (Fig. 3E). Taken together, NEDD4 was identified as a target of YAP1 in human SSCs.

### NEDD4 knockdown suppresses the proliferation and DNA synthesis and increases the apoptosis in human SSCs

As a target of YAP1, we investigated whether NEDD4 plays a role in regulating human SSC fate determinations. First, real-time PCR and Western blots were used to evaluate the knockdown efficiencies of NEDD4, which reflects the decreases in mRNA (Fig. S6A) and protein (Fig. S6B and C) levels of NEDD4 in human SSCs treated with NEDD4-siRNA, especially NEDD4-siRNA3.

**Fig. 4.**
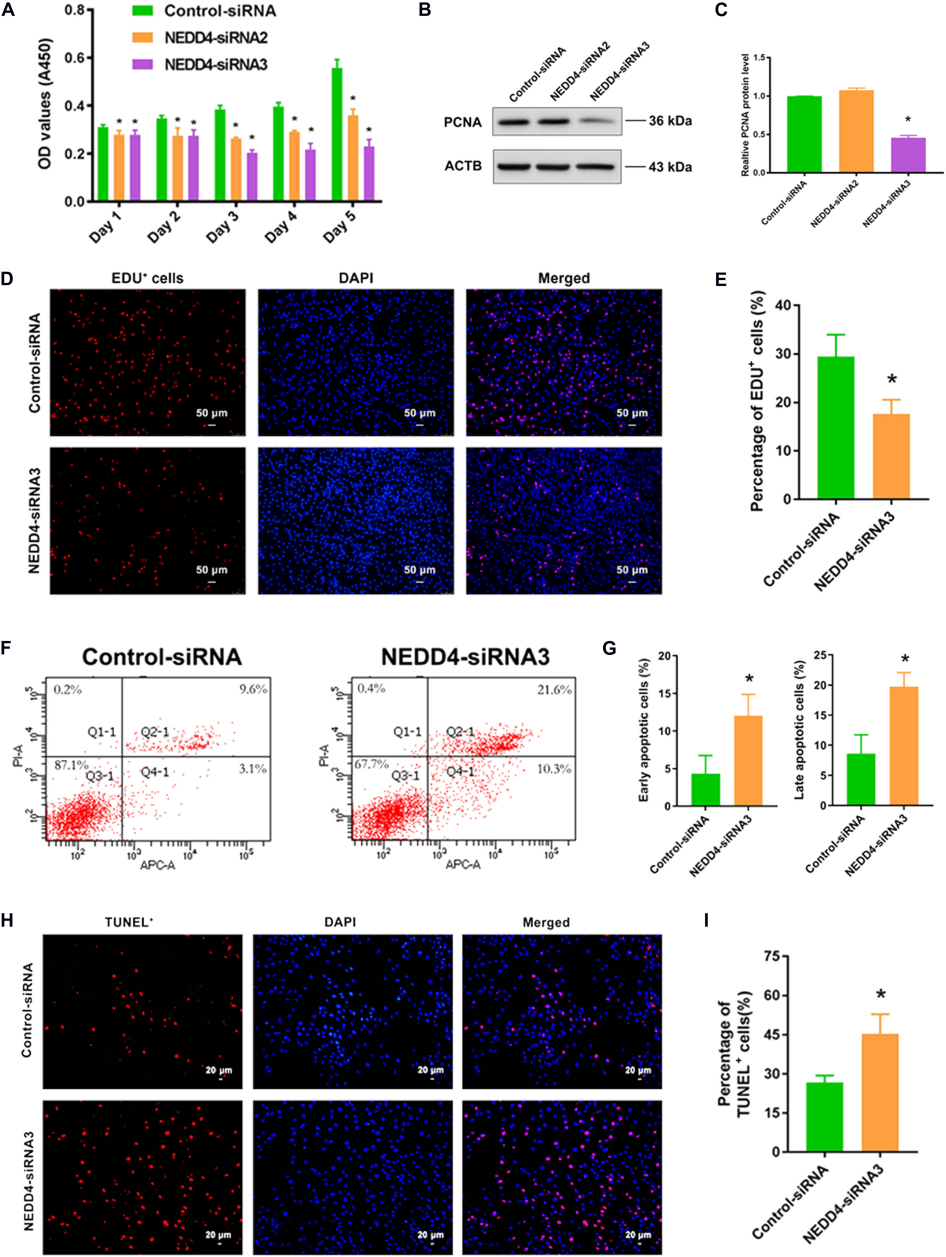
The roles of NEDD4 silencing in regulating the proliferation and apoptosis of human SSCs. (A) CCK-8 assay showed the proliferation of human SSCs after treatment of NEDD4-siRNA2 and NEDD4-siRNA3. (B and C) Western blots revealed level changes of PCNA protein in human SSCs transfected with NEDD4-siRNA2 and NEDD4-siRNA3 compared to control-siRNA. ACTB was used as a control of loading proteins. (D and E) EDU incorporation assays showed EDU-positive cells in human SSCs with control-siRNA and NEDD4-siRNA3 transfection. Scale bars, 50 μm. (F and G) Annexin V–APC/PI and flow cytometry analysis displayed the apoptosis of human SSCs affected by control-siRNA and NEDD4-siRNA3 for 48 h. (H and I) TUNEL assay showed the number of TUNEL-positive cells in human SSCs transfected by control-siRNA and NEDD4-siRNA3. Scale bars, 20 μm. *P < 0.05.

Next, CCK-8 assay displayed that cell proliferation was suppressed by NEDD4-siRNA2 and NEDD4-siRNA3 in human SSCs at 48 to 120 h after transfection (Fig. 4A). Western blots showed the decrease in PCNA protein level in human SSCs treated with NEDD4-siRNA2 and NEDD4-siRNA3 (Fig. 4B and C). EDU incorporation assays showed a notable reduction in EDU-positive cells in human SSCs upon treatment with NEDD4-siRNA3 (Fig. 4D and E). Moreover, flow cytometry and TUNEL assay were used to evaluate the apoptosis. Flow cytometry indicated that NEDD4-siRNA3 increased the apoptosis in human SSCs compared to control-siRNA (Fig. 4F and G). Similarly, proportion of TUNEL-positive cells in human SSCs was enhanced by NEDD4 silencing compared to control-siRNA (Fig. 4H and I). Collectively, these results suggest that NEDD4 knockdown reduces the proliferation and DNA synthesis of human SSCs and increases their apoptosis.

### NEDD4 overexpression counteracts the influence of YAP1 knockdown on human SSCs

**Fig. 5.**
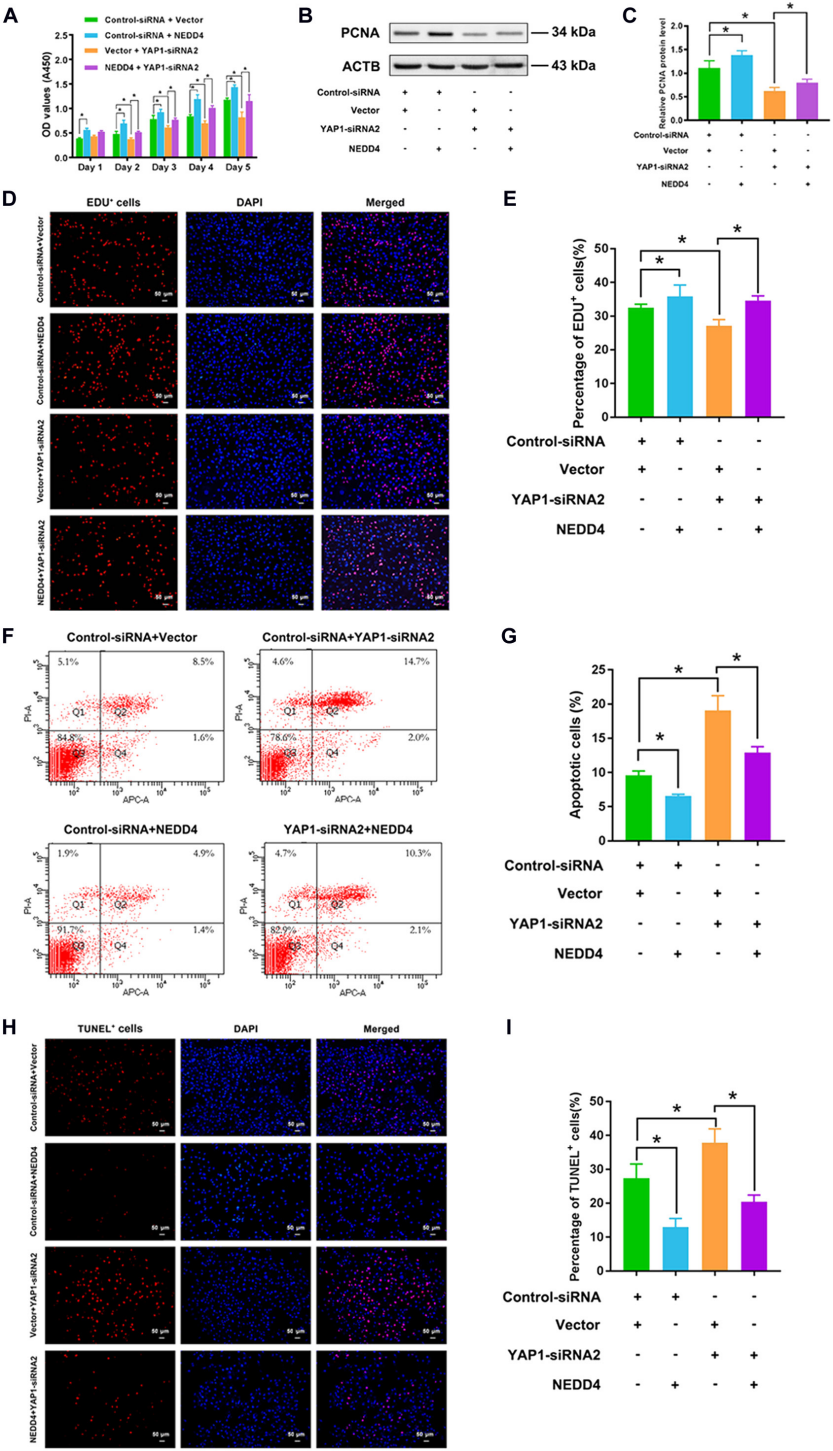
Rescue effect of NEDD4 overexpression on proliferation and apoptosis of human SSCs caused by YAP1 silencing. (A) CCK-8 assay illustrated growth in human SSCs transfected with YAP1-siRNA2 and NEDD4 overexpression. (B and C) Western blots showed the level of PCNA protein in human SSCs with YAP1 silencing and NEDD4 overexpression. (D and E) EDU incorporation assay displayed DNA replication of human SSCs with YAP1-siRNA2 and NEDD4 overexpression. Scale bars, 50 μm. (F and G) Annexin V–APC/PI and flow cytometry analysis demonstrated apoptosis of human SSCs treated with YAP1-siRNA2 and NEDD4 overexpression. (H and I) TUNEL assay showed TUNEL-positive cells in human SSCs with silencing of YAP1 and overexpression of NEDD4. Scale bars, 50 μm. *P < 0.05.

To probe whether NEDD4 mediated the influence of YAP1 knockdown on human SSCs, we performed rescue experiments. First, we constructed a NEDD4 overexpression plasmid that was verified by DNA sequencing. Real-time PCR and Western blots revealed significant increases in the mRNA (Fig. S7A) and protein (Fig. S7B and C) levels of NEDD4 in human SSCs transfected with the NEDD4 plasmid. Moreover, human SSCs overexpressing NEDD4 exhibited an increased level of PCNA protein compared to the control vector (Fig. S7B and C), which was in contrast with the effect of NEDD4 knockdown on human SSCs. Notably, NEDD4 overexpression in human SSCs reversed the reduction in proliferation caused by YAP1-siRNA2 (Fig. 5A). Similarly, NEDD4 overexpression partially rescued the decrease in PCNA protein level caused by YAP1-siRNA2 in human SSCs (Fig. 5B and C). As shown in Fig. 5D and E, NEDD4 overexpression led to an enhancement in EDU-positive cells of human SSCs, whereas YAP1-siRNA2 inhibited their DNA synthesis. NEDD4 overexpression counteracted the effect of YAP1-siRNA2 on DNA synthesis of the human SSCs. Moreover, flow cytometry (Fig. 5F and G) and TUNEL assay (Fig. 5H and I) displayed that the impact of YAP1-siRNA2 on apoptosis could be reverted by NEDD4 overexpression. Taken together, these findings imply that YAP1 silencing leads to decreases in proliferation and DNA synthesis as well as an enhancement in the apoptosis via NEDD4 in human SSCs.

### YAP1 modulates NEDD4 in human SSCs via binding to RAD21

**Fig. 6.**
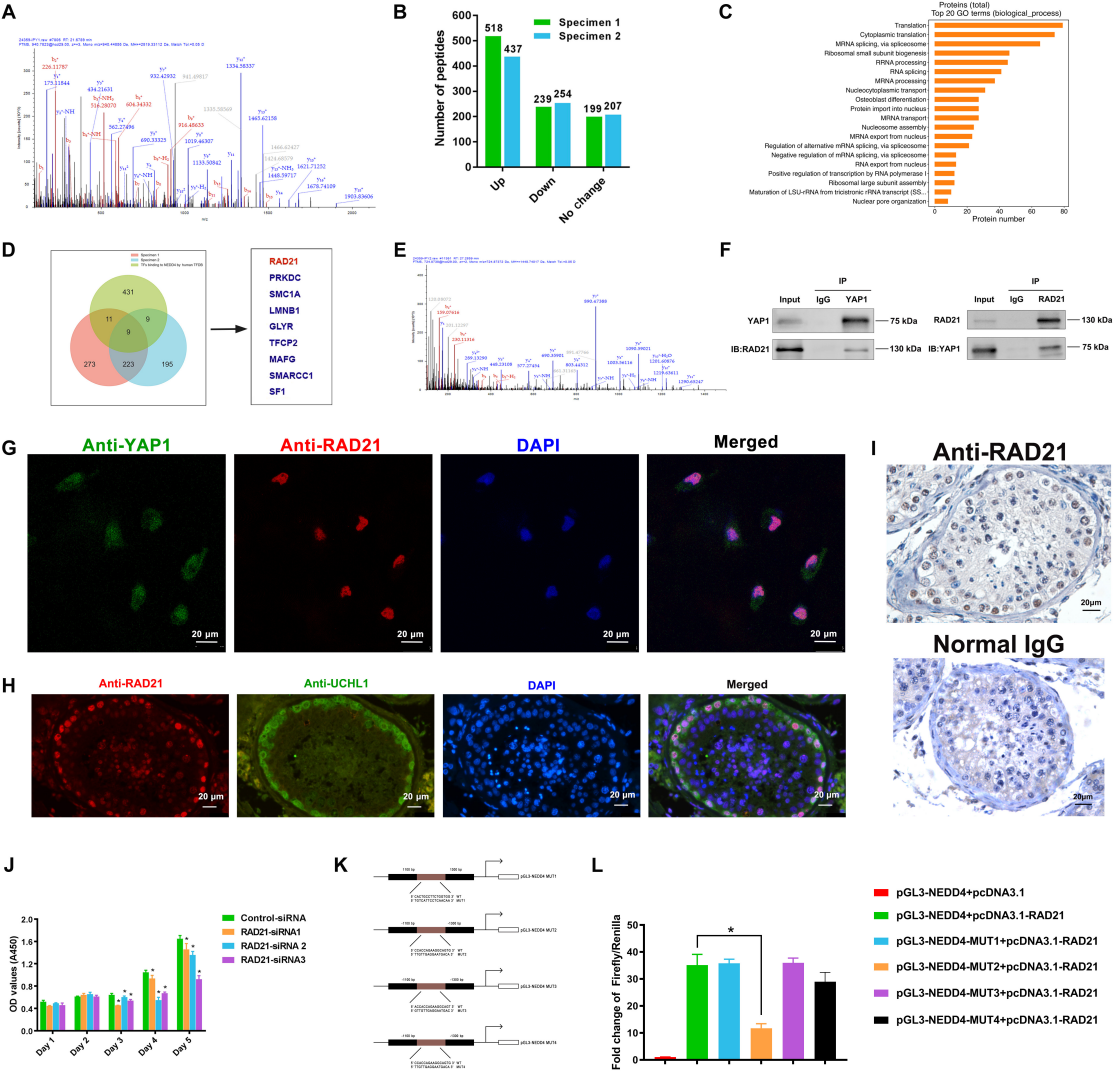
Identification of the transcription factor RAD21 as a target for YAP1 in human SSCs. (A) MS for YAP1 in human SSCs. (B) Histogram showed the number of peptides identified by Co-IP/MS in human SSCs. (C) GO analysis illustrated the top 20 enrichment functional terms. (D) Venn diagram displayed the overlaps of proteins coprecipitated by YAP1 from Co-IP/MS and predicted transcriptional factors for NEDD4. (E) MS for RAD21 in human SSCs. (F) Co-IP showed that YAP1 and RAD21 was precipitated by each other in human SSCs. (G) Double immunostaining showed that YAP1 and NEDD4 were coexpressed in the human SSCs. Scale bars, 20 μm. (H and I) Immunohistochemistry and double immunofluorescence exhibited the expression of RAD21 as well as coexpression of RAD21 and UCHL1 in normal human testicular tissues. Scale bars, 20 μm. (J) CCK-8 assay displayed growth of human SSCs treated with RAD21–siRNA1-3. (K) A schematic diagram exhibited RAD21 binding site sequence and mutation sequence in the NEDD4 promoter region. (L) Dual-luciferase reporter assays displayed the ratio of Firefly and Renilla between the WT and *NEDD4* mutations. *P < 0.05.

YAP1 is a transcriptional coactivator, and it could not bind to DNA promoter due to lacking DNA-binding domains. Thus, we speculated that YAP1 could interact with transcription factors to regulate the downstream gene NEDD4. In order to screen potential transcription factors binding to YAP1 in human SSCs, we further conducted co-immunoprecipitation (Co-IP) and mass spectrometry (MS). Our MS for YAP1 was shown in Fig. 6A, and our Co-IP and MS identified 956 and 890 proteins in human SSCs, respectively. Among them, 518/437 were up-regulated proteins, whereas 239/254 were down-regulated proteins and 199/207 were unchanged proteins (Fig. 6B). Our GO analysis showed that proteins pulled down by YAP1 were mainly enriched in the translation activity (Fig. 6C). Meanwhile, the promoter region of NEDD4 was determined to be from transcription start site (TSS) to position TSS+ 2,000 base pairs in human chromosome 15 (Chr15: 55993612-55995612, GRCh38.p14). Next, human transcription factor database was employed to predict the potential transcription factors binding to the promoter of NEDD4. Subsequently, we took the intersection of both the up-regulated proteins for Co-IP/MS and the potential transcription factors of NEDD4, and a Venn diagram was plotted. There were 9 transcription factors, including RAD21 (RAD21 cohesin complex component), PRKDC, SMC1A, LMNB1, GLYR, TFCP2, MAFG, SMARCC1, and SF1 (Fig. 6D). To uncover the potential binding proteins, we screened the above transcription factors based on the localization, expression, and bioinformatics analysis with Cistrome Data Brower. Notably, RAD21 was identified as the potential intermediate factor that links YAP1 and NEDD4 in human SSCs. Our MS for RAD21 was shown in Fig. 6E.

In order to demonstrate the interaction between YAP1 and RAD21, we performed the Co-IP analysis. Endogenous Co-IP showed that RAD21 was co-precipitated with YAP1 (Fig. 6F). Double immunostaining revealed that YAP1 (green fluorescence, nuclei-cytoplasm) and RAD21 (red fluorescence, nuclei) are coexpressed in the human SSCs (Fig. 6G). Intriguingly, immunohistochemistry of human testes displayed that RAD21 was specially located in the nuclei of human primary SSCs along with the basement membrane of seminiferous tubules rather than other male germ cells in human testicular tissues (Fig. 6I, upper panel). Notably, double immunostaining demonstrated that RAD21 was co-expressed with UCHL1, a marker for human SSCs, in primary SSCs of human testes (Fig. 6H). RAD21 has been known to be involved in DNA damage repair. We designed 3 RAD21-siRNAs that could effectively knock down the mRNA (Fig. S8A) and protein (Fig. S8B and C) levels of RAD21. CCK-8 assay exhibited that RAD21 silencing inhibited the proliferation of human SSCs (Fig. 6J). Considered together, these findings imply that RAD21 exerts a significant role in maintaining the stemness of human SSCs mediated by YAP1.

Next, we analyzed NEDD4 promoter sequences using the hTFtarget database to predict transcription factor binding sites. We found that multiple binding sites in the NEDD4 promoter sequences may bind to RAD21 (Table S3). We chose 4 binding sites with high scores to perform dual-luciferase reporter assay (Fig. 6K). Compared to control plasmid, the NEDD4 promoter activity was enhanced by RAD21 in human SSCs (Fig. 6L). When site 2 was mutated, NEDD4 activity enhanced by RAD21 was reduced in these cells (Fig. 6L). Collectively, these data suggest that YAP1 binds to RAD21 and targets NEDD4 to mediate the proliferation, DNA synthesis, and apoptosis of human SSCs.

### *Yap1* cKO inhibits proliferation and enhances apoptosis of SSCs in vivo

**Fig. 7.**
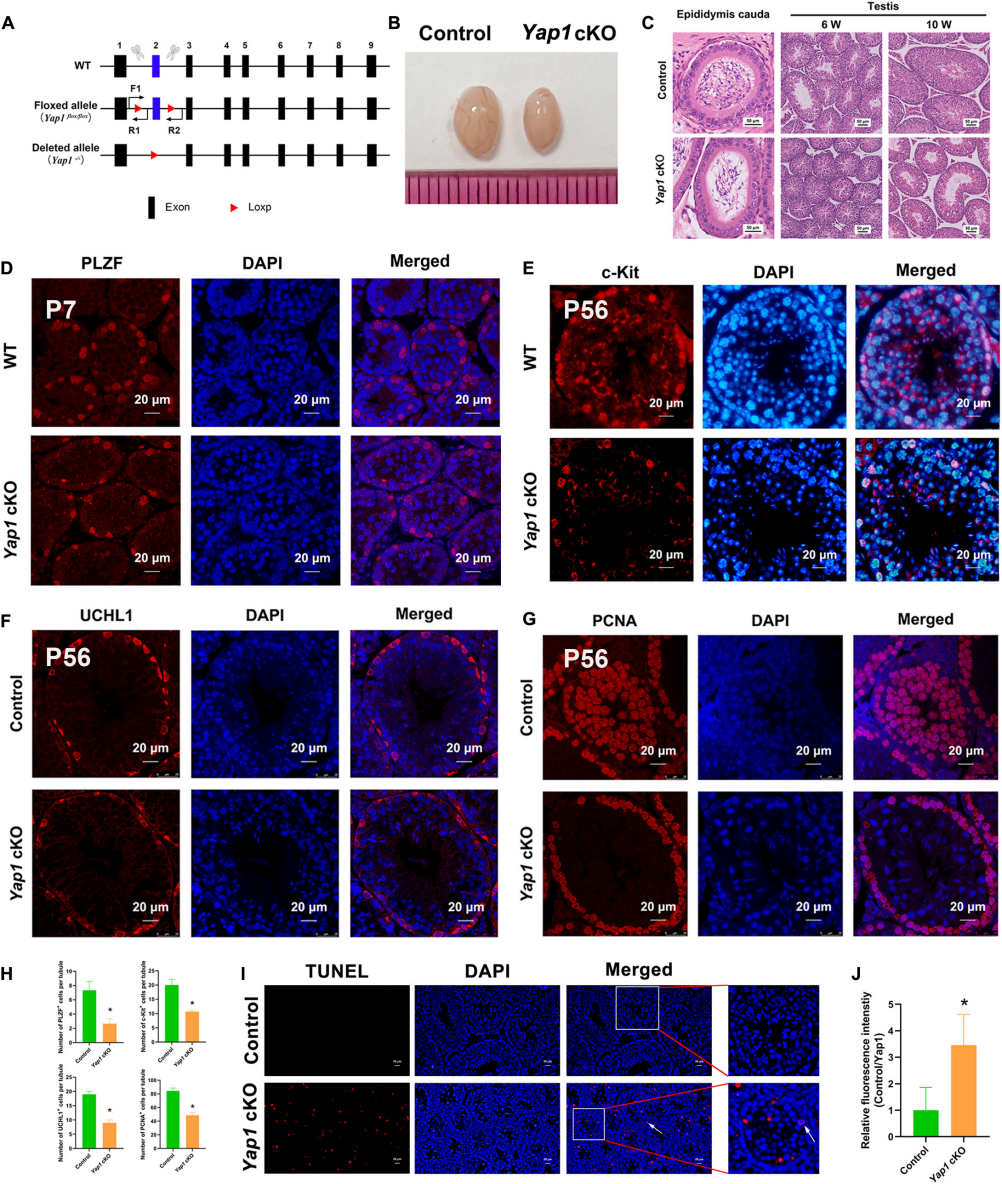
Yap1 cKO inhibits proliferation and enhances apoptosis of SSCs in vivo. (A) Hybrid scheme was used to generate the *Yap1* flox/Δ;Stra8-Cre (*Yap1* cKO) mice. (B) Gross morphology of testes from control WT mice and *Yap1* cKO mice at 10 weeks. (C) H&E staining of testes and epididymis from control WT mice and *Yap1* cKO mice. (D to H) Immunohistochemistry revealed the expression profiles of PLZF (D), c-Kit (E), UCHL1 (F), PCNA (G), and (H) statistical histograms displaying the number of positive cells in each seminiferous tubule and in the seminiferous tubules of WT mice and *Yap1* cKO mice. Scale bars, 20 μm. (I and J) TUNEL assay showed the number of TUNEL-positive cells in seminiferous tubules of WT mice (upper panel) and *Yap1* cKO mice (lower panel).

To probe the function of YAP1 in controlling SSCs and spermatogenesis in vivo, we generated *Yap1* cKO mice by crossing *Yap1* floxed mice and *Stra8-EGFP* Cre mice. For the *Yap1* cKO mice, the exon 2 of *Yap1* was deleted (Fig. 7A). Eventually, *Yap1* cKO mice was successfully generated as demonstrated by the absence of *Yap1* transcription (Fig. S9A) and Yap1 protein (Fig. S9B) in testes of mice as shown by reverse transcription PCR (RT-PCR) and Western blots, respectively. The adult testis/weight ratio in the *Yap1* cKO mice was lower than that in the WT mice (Fig. 7B and Fig. S9C). Meanwhile, in adult *Yap1* cKO mice, the count of sperm in the cauda epididymis was remarkably fewer compared to control mice (Fig. 7C, lower and left panels). There was almost no active spermatogenesis in the seminiferous tubules of *Yap1* cKO mice at 6 weeks (Fig. 7C, lower and middle panels). Similarly, H&E staining of the testicular sections in *Yap1* cKO mice revealed a decrease in spermatozoa count at 10 weeks (Fig. 7C, lower and right panels). Together, these data indicate that *Yap1* cKO results in spermatogenesis disorder.

To explore the impact of Yap1 knockout on the fate determinations of SSCs in *Yap1* cKO mice, we determined the expression of SSC markers (PLZF and UCHL1) and spermatogonial differentiation hallmark (c-Kit) utilizing immunofluorescence. Compared with control WT mice, the percentages of PLZF-positive cells (Fig. 7D and H) at 7 days after birth (P7), c-Kit-positive cells (Fig. 7E and H) at P56, and UCHL1-positive cells (Fig. 7F and H) in *Yap1* cKO mice were lower. Meanwhile, we detected proliferation and apoptosis of SSCs using immunofluorescence and TUNEL assay. The proportion of PCNA-positive cells (Fig. 7G and H) was obviously decreased in the seminiferous tubules of *Yap1* cKO mice, whereas the percentages of TUNEL-positive cells were dramatically increased in *Yap1* cKO mice (Fig. 7I and J). Taken together, these data implicate that *Yap1* knockout leads to notable reduction in the proliferation and a remarkable increase in apoptosis of SSCs in vivo.

### *Yap1* cKO causes oligoasthenoteratospermia in mice

**Fig. 8.**
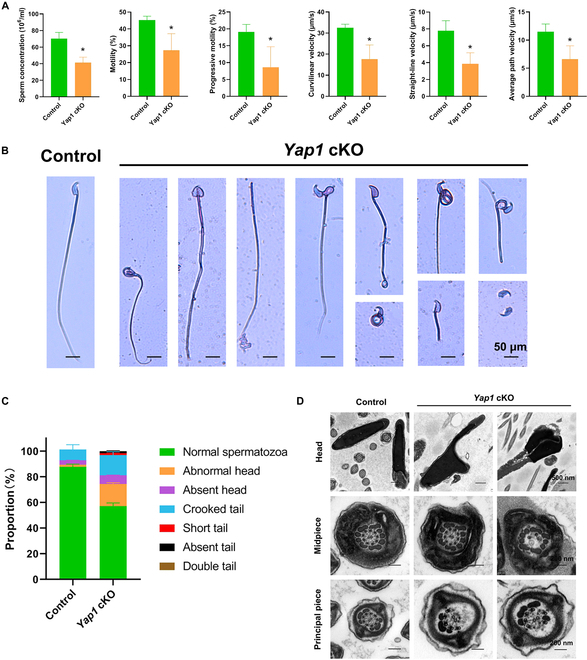
The functions of YAP1 in mediating spermatogenesis in vivo. (A) Sperm analysis in control WT mice and *Yap1* cKO mice by CASA. (B and C) Sperm morphology examined by Papanicolaou staining and percentages of abnormal sperm in the *Yap1* cKO mice and control WT mice. Scale bars, 50 μm. (D) Ultrastructure of sperm detected by TEM. Scale bars, 500 nm (head) and 200 nm (tail).

We further analyzed the roles of Yap1 in mediating late stage of spermatogenesis in *Yap1* cKO mice. Interestingly, computer-assisted sperm analysis (CASA) revealed significant decreases in sperm count and sperm motility parameters, e.g., progressive motility, curvilinear velocity, straight-line velocity, and average path velocity, in the cauda epididymis of *Yap1* cKO mice (Fig. 8A). Sperm morphology was assessed by Papanicolaou staining. As seen in Fig. 8B and C, there were a number of sperm with aberrant morphology, including abnormal head formations, acephalic sperm, bent tails, shorted tails, and absent tails. The ultrastructural analysis of epididymal sperm was performed with transmission electron microscopy (TEM). In the midpiece of most flagella of the *Yap1* cKO mice, a disordered structure of mitochondrial sheath was observed (Fig. 8D). In the principal piece of numerous sperm tails, we found the disorganized microtubule doublets (OD) and outer dense fiber (ODF) (Fig. 8D). Collectively, these findings indicate that *Yap1* depletion causes oligoasthenoteratospermia in mice and Yap1 is involved in the regulation of spermiogenesis.

## Discussion

SSCs are the initiating cells for spermatogenesis, and the fate determinations of SSCs are regulated by key genes and epigenetic factors [4,5]. Nevertheless, the molecular mechanisms underlying human SSC fate determinations and spermatogenesis disorders remain largely unknown. In this study, we investigated an important roles of YAP1 in controlling spermatogenesis and the association of its abnormality with NOA patients. We found that YAP1 levels were obviously lower in the testicular tissues of NOA patients than in normal males and that these patients had have a higher frequency of *YAP1* SNVs using WES analysis. Our finding suggests that YAP1 dysfunction may be linked to the development of NOA. Furthermore, we demonstrated that YAP1 could interact with the transcription factor RAD21 to control the self-renewal (the proliferation and DNA synthesis) and the apoptosis of human SSCs via targeting NEDD4.

YAP1 is a crucial downstream effector of the Hippo signaling pathway, and it is regulated by nucleocytoplasmic shuttling [25]. In this study, we revealed that YAP1 was mainly located at the nuclei of both human SSC line and human primary SSCs. This cellular localization of YAP1 in human SSCs was consistent with other cell types, e.g., granulosa cells [30], hepatocytes [31], and epidermal stem cells [32]. Using bioinformatics predictions and protein stability experiments, we demonstrated that the S227L variant of *YAP1* affected the stability of the YAP1 protein. Furthermore, we showed that YAP1 silencing led to the decrease in proliferation of human SSCs and an increase of their apoptosis and that YAP1 S227L variant had the same functions. These results underscore the important role of YAP1 and its variants in regulating the fate determinations of human SSCs in vitro. In addition, xenotransplantation model was used to examine the influence of YAP1 on SSC fate determinations. We found that YAP1 silencing suppressed the self-renewal of human SSCs in vivo*.* In previous studies, YAP1 plays a crucial role in governing the fate determinations of various stem cell populations [33], e.g., muscle stem cells [34], retinal progenitor cells [35], and neural progenitors [36]. Yap1/Taz signaling in mesenchymal progenitor indirectly enhances the proliferation of muscle stem cells [34]. At a high cell density, YAP1 also promotes their proliferation in porcine muscle stem cells [37]. In bone marrow mesenchymal stem cells (BMSCs), when Yap1 is activated, the mitochondrial pathway enhances their apoptosis. In the hypoxia microenvironment, hypoxia-inducible factor 1α (HIF1α) binds to the dephosphorylated Yap1 in the cell nuclei to form a complex, which transactivates the target genes responsible for the survival of BMSCs and thus inhibits the apoptosis [38]. In the present study, we have demonstrated that YAP1 silencing results in decreases in the self-renewal as well as an enhancement in the apoptosis of human SSCs in vivo and in vitro. This study highlights an essential role of YAP1 in male reproduction and its significance in the pathogenesis of NOA. Therefore, YAP1 might have significant applications in both reproductive medicine and regenerative medicine.

Nuclear YAP1, the active form of YAP1, acts as a transcription cofactor by binding to transcription factors [39], e.g., TEAD and Smad, which regulates downstream target genes. In this study, NEDD4 was recognized as a downstream target gene. Nevertheless, NEDD4 is a ubiquitinated protein and it cannot bind to YAP1 directly. Therefore, we performed Co-IP/MS with anti-YAP1 and identified transcription factors regulating NEDD4 through bioinformatics tools online. Although TEAD, a known transcription factor of YAP1, has been discovered via Co-IP/MS in human SSCs, it was not a direct transcription factor of NEDD4. Double immunostaining and overexpression or silencing of TEAD could be employed to demonstrate whether there is an interaction of NEDD4 and TEAD. Interestingly, RAD21 was identified by us as an intermediate factor linking YAP1 and NEDD4 in human SSCs, as shown by our Co-IP and YAP1 and NEDD4 silencing. Notably, our double immunostaining revealed that RAD21 was highly coexpressed with UCHL1 in human SSCs within the seminiferous tubules of human testes, which reflects the significance of RAD21 in governing fate determinations of human SSCs. RAD21 is one of the components of the cohesin complex, including SMC1A, SMC3, REC8, and STAG, which mediates the sister chromosome segregation, recombination, DNA replication, and transcriptional regulation in mitosis and meiosis [40–43]. Previous studies have reported the key roles of cohesin, especially RAD21, in the regulation of transcriptional processes and reproduction [44–46]. RAD21 is specifically expressed in testis and ovary of Chinese mitten crab *Eriocheir sinensis* [47]. The mutations of STAG3 cohesin cause male infertility via meiotic arrest [48]. The absence of REC8 cohesion leads to germ cell failure and female mouse sterility [49]. Significantly, we have identified, for the first time, that RAD21 was an intermediate factor between YAP1 and NEDD4 in mediating the mitosis of human SSCs. This was consistent with a recent study showing that the RAD21–YAP complex modulates immune response in ovarian cancer [50].

Notably, we constructed cKO *Yap1* mice that assumed mitosis arrest of SSCs, abnormal sperm morphology, and poor sperm progressive motility, which indicates that YAP1 affects not only the division of SSCs but also spermiogenesis and spermatid function. Double deficiency of Mst1 and Mst2, upstream effectors of YAP1, has been observed in male infertility [28], and the Hippo pathway may affect male reproduction development, except for YAP1, MST1, and MST2. Furthermore, YAP1 in oocytes is associated with the zygotic genome activation. Maternal *Yap1* knockout embryos exhibit a prolonged 2-cell stage and develop into the 4-cell stage at a slower pace than the WT [51,52]. It is interesting to further explore the impact of LAST1/2 on reproductive function.

## Conclusion

**Fig. 9.**
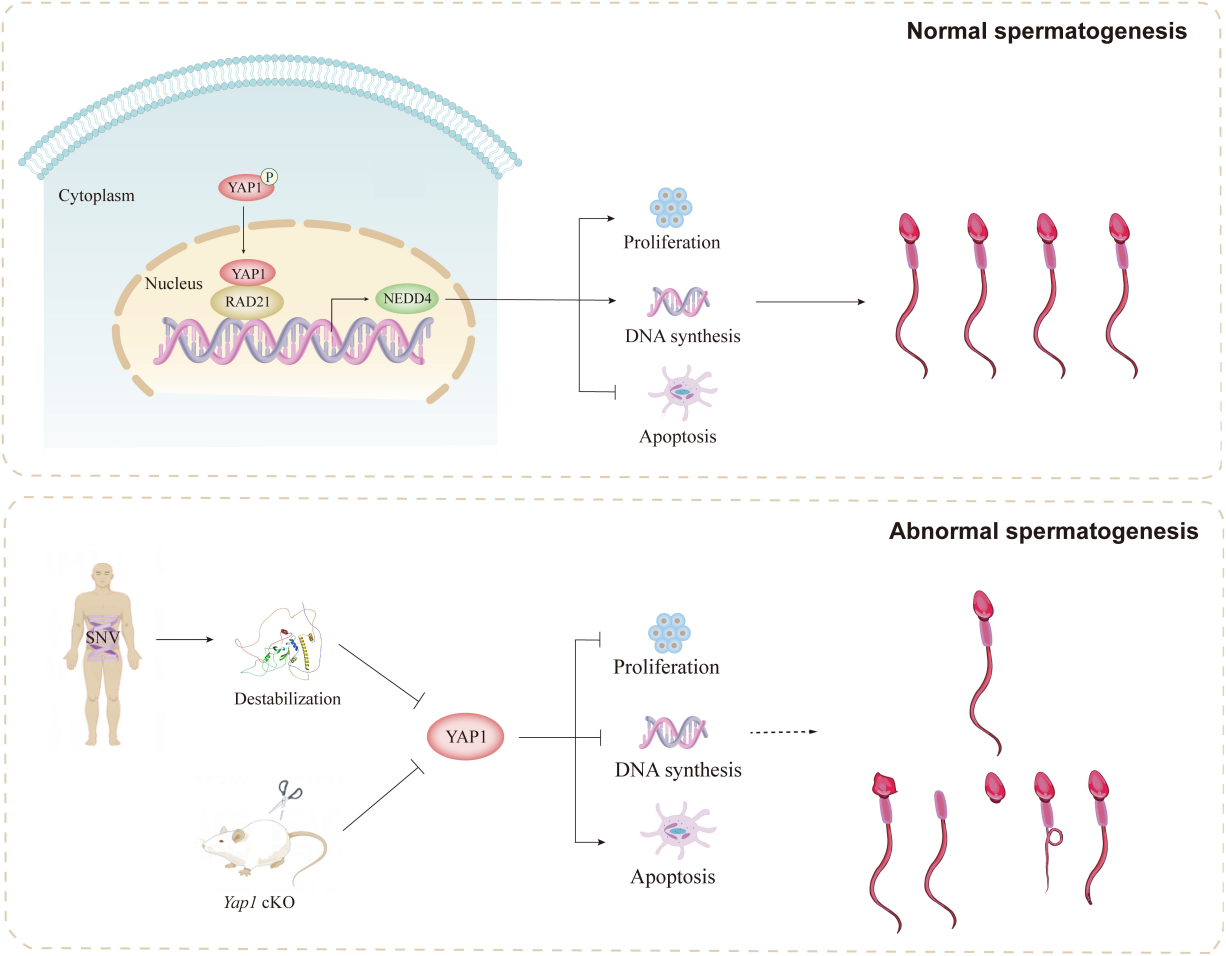
The functions and mechanisms of YAP1 and its dysfunction in mediating fate determinations of SSCs and spermatogenesis. YAP1 interacts with RAD21 to control the self-renewal and apoptosis of human SSCs through targeting NEDD4. YAP1 SNVs and/or lower level is associated with risk of NOA. *Yap1* cKO results in abnormal spermatogenesis and male infertility.

In summary, we have found that YAP1 is localized predominantly at the nuclei of human SSCs in human testes. We further reveal that YAP1 can interact with the transcription factor RAD21 to control the self-renewal (proliferation and DNA synthesis) and the apoptosis of human SSCs via targeting NEDD4. YAP1 SNVs and/or lower level might be associated with the risk of NOA. Notably, *Yap1* cKO leads to mitosis arrest of SSCs, the decreases in the number and motility of mature sperm, abnormal sperm morphology, and an increase of apoptosis in male germ cells. Collectively, we are the first to demonstrate that the YAP1/RAD21/NEDD4 pathway mediates the fate determinations of human SSCs and abnormality or mutations of YAP1 are associated with male infertility (Fig. 9). As such, this study presents a novel genetic regulatory network that controls the fate determinations of human SSCs and contributes to the understanding of NOA etiology. Notably, this study identifies new biomarkers for genetic intervention in patients with male infertility.

## Methods

### Acquirement of human testicular tissues

Testicular tissues were collected from patients diagnosed with OA and NOA undergoing testicular biopsy or surgical resection at The Third Xiangya Hospital of Central South University and Hunan Cancer Hospital. The tissues were rinsed with phosphate-buffered solution (PBS) supplemented with 4% penicillin and streptomycin. Some testicular tissues were preserved in Bouin’s fixative (Servicebio) for paraffin-embedded sections, while the remaining tissues were utilized for RNA and protein extraction. Human testicular tissues were used for research only and approved by the Ethics Committee of Hunan Normal University.

### Culture of human SSC line

Human SSC line was cultured in Dulbecco’s modified Eagle’s medium (DMEM)/F12 (Gibco), supplemented with 10% fetal bovine serum (FBS) (Gibco), 100 units/ml penicillin (Gibco), and 100 g/ml streptomycin (Gibco) in a humidified atmosphere incubator of 5% CO_2_ at 34 °C. This cell line was characterized using RT-PCR and immunocytochemistry to identify as human primary SSCs.

### Xenotransplantation of human SSCs to testes of mice

BALB/c male mice at 6 weeks old were obtained from Hunan Slake Jingda Experimental Animal Co. Ltd. (Changsha, China), and they were housed in the specific pathogen-free (SPF) animal facility. Busulfan (40 mg/kg body weight) was intraperitoneally injected into mice to remove male germ cells. After 4 weeks for busulfan injection, human SSC line transfected with YAP1-siRNA2 or control-siRNA was transplanted into seminiferous tubules of mice pursuant to the previously described method [19]. Four weeks after cell transplantation, testicular tissues were obtained from recipient mice for further studies. The animal experiments were ethically approved by the Ethics Committee of Hunan Normal University.

### Generation of *Yap1* cKO mice

*Yap1* floxed mice were provided by J. Wu from Xiamen University, and *Stra8-EGFP*-Cre mice with C57BL/6J background were obtained from Saiye Biotechnology Co. Ltd. (Jiangsu, China). First, *Yap1^flox/wt^* mice were bred with each other to obtain *Yap1^flox/flox^* mice. Next, the *Yap1* homozygous mice were crossed with *Stra8*-Cre mice to generate *Yap1^flox/wt^*;*Stra8*-Cre mice that were then crossed with *Yap1^flox/flox^* mice to acquire *Yap1 ^flox/^*;*Stra8*-Cre mice (designated as *Yap1* cKO mice). Genotyping of *Yap1* cKO mice was conducted via PCR using genomic DNA extracted from tail samples. The gene primers for genotyping were shown in Table S6. The animal experiments were conducted in accordance with the ethical standards and guidelines approved by the Ethics Committee of Hunan Normal University.

### Immunocytochemistry and immunohistochemistry

For immunocytochemistry, human SSCs treated with different siRNAs were harvested with trypsinization and resuspended in PBS. These cells were attached to the glass slides using cytospin (Cence) at 1,700 rpm for 1 min, and fixation was performed with 4% paraformaldehyde (PFA) for 15 min. After cold PBS washing, cells underwent 15 min of permeabilization at room temperature with 1% Triton. Subsequently, they were blocked with 5% bovine serum albumin for 1 h at room temperature, followed by overnight incubation with primary antibodies at 4 °C and 1-h incubation with fluorescent secondary antibodies at room temperature. Cell nuclei were stained with 4′,6-diamidino-2-phenylindole (DAPI), and the images were acquired using a confocal microscope (Leica TCS SP8 SR) or fluorescence microscope (Leica DM3000LED).

For immunohistochemistry, paraffin sections of testes were dewaxed at 65 °C for 90 min and dehydrated with gradient alcohol. Antigen retrieval was conducted through a 20-min boiling process in 1× sodium citrate buffer, and blocking of endogenous peroxidase was conducted with 3% hydrogen peroxide. The procedures of blocking, antibody incubation, and imaging capture were similar to the method as described in immunocytochemistry. The detailed information on the antibodies used in immunocytochemistry and immunohistochemistry, including the dilution and concentrations, was shown in Table S4.

### Transfection of siRNAs and plasmids into human SSCs

The siRNAs for silencing PDK1, YAP1, NEDD4, and RAD21 were synthesized by GenePharma, while a nontargeting control-siRNA was employed as the negative control. The sequences of all siRNAs were listed in Table S5. The NEDD4-overexpressing plasmid carrying a red fluorescent (Cherry) was constructed by Shanghai Jikai Gene Technology Company, while empty vector was utilized for the negative control. According to the manufacturer’s protocol, cells were transfected with siRNAs or plasmids using Lipofectamine 3000 (Invitrogen). The relative gene and protein levels were analyzed via real-time PCR and Western blots at 48 h after transfection, respectively.

### Total RNA isolation, RT-PCR, and real-time PCR

Total RNA was extracted using Trizol reagent (Invitrogen) in terms of the manufacturer’s protocol. Subsequently, the concentration and quality of total RNA were determined with NanoDrop One (Thermo Fisher Scientific). Next, Evo M-MLV RT Premix (Accurate Biology) was used to reverse transcribe the total RNA into cDNA, which was then used for PCR and real-time PCR of genes, respectively. *ACTB*, a housekeeping gene, was chosen to detect the loading RNA, while negative control did not contain any cDNA.

PCR was carried out in accordance with the protocol as described previously [53], and the reaction conditions comprised an initial denaturation at 95 °C for 5 min and followed by 35 cycles of denaturation at 95 °C for 30 s, annealing at 60 °C for 30 s, and elongation at 72 °C for 5 min. The PCR products were separated on 2% agarose gels stained with GoldView.

Real-time PCR was conducted utilizing 2× SYBR Green Premix Pro Taq HS qPCR Kit (Accurate Biology) in 20-μl reaction via the Bio-Rad CFX system. The gene expression was normalized to *ACTB* using the 2^−ΔΔCT^ method. The sequences of gene primers synthesized by Shenggong Biotech were listed in Table S6.

### Isolation of YAP1 cytosolic and nuclear proteins, and Western blots

Protein extraction was performed by radioimmunoprecipitation assay (RIPA) supplemented with 0.1 mM phenylmethylsulfonyl fluoride and protease inhibitor cocktail (Thermo Fisher Scientific). The cytoplasmic and nuclear proteins were extracted using a nucleocytoplasmic protein extraction kit (Beyotime). The protein supernatant was combined with 5× sodium dodecyl sulfate (SDS)-loading buffer and denatured by boiling at 99 °C for 10 min.

Following separation via 10% SDS-polyacrylamide gel electrophoresis gel electrophoresis, proteins were transferred onto polyvinylidene difluoride membranes with 0.45-μm pore sizes (Millipore). After blocking with QuickBlock Blocking Buffer (Beyotime) for 30 min, primary antibodies were applied and incubated overnight at 4 °C. Horseradish peroxidase-conjugated secondary antibodies were used, and protein bands were visualized with enhanced chemiluminescence reagents and analyzed using ImageJ software. The detailed information on antibody dilution concentrations and specifics was shown in Table S7.

### Protein stability assay

Cells were seeded in 6-well plates, and CHX was added into each well at a concentration of 200 μg/ml to block new protein synthesis. Cells were harvested at 0, 6, and 12 h after CHX treatment. Total protein was extracted using RIPA lysis buffer. Protein samples were used for Western blotting pursuant to the method as described above.

### Co-immunoprecipitation (Co-IP)

The Pierce IP/Co-IP kit (Thermo Fisher Scientific) protocol was utilized for proteins’ interaction assay. Human SSCs were cultured in 10-cm dishes until they reached 85% confluence, and they were lysed using 500 μl of binging/washing buffer. The cell lysates were added with 10 μg of antibodies, including YAP1 or RAD21, and incubated overnight at 4 °C. Next, the antibody–lysate complexes were subjected to IP using 25 μl of protein A/G beads and constantly rotated for 2 h at room temperature. The magnetic rack was employed to gather the magnetic bead–immune complex, and washing buffer and rinsing with ultrapure water were conducted. Subsequently, the antigen–antibody–bead complexes were eluted with elution buffer and denatured for Western blots.

### Whole-exome sequencing (WES)

DNA extraction kit (QIAGEN) was utilized to extract genomic DNA from peripheral blood of OA and NOA patients according to the manufacturer’s instructions. DNA concentration and purity were evaluated, and construction of the whole exome library was conducted, followed by sequencing on the Illumina HiSeq 2000 or NovaSeq 6000 sequencing platforms (Illumina). The raw reads underwent alignment to GRCh37/hg19 using the Burrows–Wheeler Aligner. Genetic variations, e.g., SNVs, deletions, and small insertions, were analyzed, annotated, and filtered by various public databases and in silico tools, including 1000G, gnomAD, ExAC, SIFT, PolyPhen-2, and MutationTaster.

### CCK-8 assays

Human SSCs were seeded into 96-well plates, with subsequent treatment with various kinds of siRNAs or control-siRNA. CCK-8 assays were conducted using CCK-8 (Dojindo) after siRNA transfection from 24 to 120 h. Following the manufacturer’s guidelines, each well was received 10 μl of CCK-8 solution and 90 μl of DMEM/F12 with a 4-h incubation at 34 °C. Absorbance measurements were performed at a wavelength of 450 nm.

### EDU incorporation assays

The Cell-Light EDU Apollo Kit (Ribobio) was utilized to conduct EDU incorporation assays pursuant to the manufacturer’s instructions. Briefly, EDU solution was diluted at a ratio of 1:2,000 with DMEM/F12 medium and added into wells of human SSCs (100 μl/well) for overnight. The cells were subjected to fixation with 4% PFA for 30 min at room temperature and followed by neutralization with 2 mg/ml glycine for 5 min and permeabilization with 0.5% Triton X-100 for 10 min. Staining of EDU was conducted by incubating the cells with Apollo staining buffer in the dark for 30 min. After staining the nuclear DNA with DAPI or Hoechst 33342, the images were captured under a fluorescence microscope (Olympus). The ratio of EDU-positive cells was calculated from at least 500 cells with ImageJ.

### Flow cytometry with APC Annexin V/PI staining

Flow cytometry with APC Annexin V/PI staining was used to analyze apoptosis in human SSCs treated with different siRNAs in terms of a previously described method [20]. Briefly, human SSCs in 6-well plates were digested with trypsin without EDTA after siRNA transfection for 48 h. The cells were harvested, rinsed with PBS, centrifuged, and then suspended in 100 μl of Annexin V Binding Buffer (BioLegend). Next, 5 μl of APC Annexin V and 10 μl of PI (BioLegend) were added to cell suspension and followed by a 15-min incubation at room temperature in the dark. Flow cytometry (BD Biosciences) was utilized to quantify apoptotic cells.

### RNA sequencing

Human SSCs were received 24-h treatment of either control-siRNA or YAP1-siRNAs and followed by total RNA extraction for RNA sequencing. RNA quality was assessed with the Agilent Bioanalyzer, while construction and sequencing of RNA libraries were conducted using the Illumina HiSeq X Ten platform. For data analysis, raw reads were performed for quality control, aligned, and annotated, and FPKM values were calculated using cufflinks2 software. The count of each sample was normalized using DESeq software. The fold changes were calculated, and a significance test was conducted to identify the DEGs based on the criteria of fold change >2.0 or <0.5 and *P* < 0.05.

### Co-IP/mass spectrometry (MS)

IP with anti-PDK1, anti-YAP1, anti-RAD21 antibodies and normal immunoglobulin G (IgG) (Table S7) were used for MS. The magnetic bead samples were eluted, reduced with dithiothreitol, alkylated with iodoacetamide, and digested into polypeptides with trypsin. After desalting, the polypeptides were measured on a liquid chromatography–MS system comprising UltiMate 3000 RSLCnano (Thermo Fisher Scientific) and Q-Exactive Plus (Thermo Fisher Scientific). Raw data were analyzed with ProteomeDiscover software and identified via the UniProt-Human database. The identified proteins were employed for subsequent MS analysis.

### Double-luciferase reporter assay

The plasmids of pGL3-NEDD4, pcDNA3.1, pcDNA3.1-RAD21, pGL3-NEDD4 MUT1, pGL3-NEDD4 MUT2, pGL3-NEDD4 MUT3, and pGL3-NEDD4 MUT4 (Miaoling Biology, China) were transfected into human SSCs using Lipofectamine 3000. After 48 h of transfection, the cells were treated and subjected to luciferase activity detection using the Dual-Glo Luciferase Reporter Assay System (E2920, Promega) by the tube luminometer (Berthold, Germany).

### Sperm analysis

The cauda epididymis was isolated from *Yap1* cKO mice, sectioned into multiple pieces, and immersed in preheated G-IVF Plus (Vitrolife, Gothenburg, Sweden, LOT509784) at 34 °C for 30 min. In total, 10 μl of the supernatant was used to detect sperm motility by the CASA system. For estimation of sperm morphology, 5 μl of the supernatant was spread over slides, dried, and fixed in 95% ethanol for the modified Papanicolaou staining.

After fixation with 2.5% glutaraldehyde and osmium tetroxide, the cauda epididymis underwent dehydration in acetone followed by embedding in Epon 812. The morphology of sperm from *Yap1* cKO mice was observed under a light microscope after staining the semithin sections with methylene blue. After being stained with uranyl acetate and lead citrate, the ultrathin sections underwent ultrastructure examination under a JEM-1400-FLASH TEM.

### Statistical analyses

SPSS (IBM SPSS Statistics, version 23.0) was utilized for all statistical analyses. The mean ± standard deviation was calculated from data from 3 independent experiments. One-way analysis of variance (ANOVA) was conducted to assess the significance between groups.

## Data Availability

All data of this study are available from the corresponding author upon request.

## References

[1] Agarwal A, Baskaran S, Parekh N, Cho CL, Henkel R, Vij S, Arafa M, Panner Selvam MK, Shah R. Male infertility. Lancet. 2021;397(10271):319–333. 33308486 10.1016/S0140-6736(20)32667-2

[2] Sun H, Gong TT, Jiang YT, Zhang S, Zhao YH, Wu QJ. Global, regional, and national prevalence and disability-adjusted life-years for infertility in 195 countries and territories, 1990-2017: Results from a global burden of disease study, 2017. Aging. 2019;11(23):10952–10991. 31790362 10.18632/aging.102497 PMC6932903

[3] Jarow JP, Espeland MA, Lipshultz LI. Evaluation of the azoospermic patient. J Urol. 1989;142(1):62–65. 2499695 10.1016/s0022-5347(17)38662-7

[4] Du L, Chen W, Cheng Z, Wu S, He J, Han L, He Z, Qin W. Novel gene regulation in normal and abnormal spermatogenesis. Cells. 2021;10(3):666. 33802813 10.3390/cells10030666 PMC8002376

[5] Wang M, Liu X, Chang G, Chen Y, An G, Yan L, Gao S, Xu Y, Cui Y, Dong J, et al. Single-cell RNA sequencing analysis reveals sequential cell fate transition during human spermatogenesis. Cell Stem Cell. 2018;23(4):599–614.e4. 30174296 10.1016/j.stem.2018.08.007

[6] Zhao J, Lu P, Wan C, Huang Y, Cui M, Yang X, Hu Y, Zheng Y, Dong J, Wang M, et al. Cell-fate transition and determination analysis of mouse male germ cells throughout development. Nat Commun. 2021;12(1):6839. 34824237 10.1038/s41467-021-27172-0 PMC8617176

[7] Helsel AR, Yang QE, Oatley MJ, Lord T, Sablitzky F, Oatley JM. ID4 levels dictate the stem cell state in mouse spermatogonia. Development. 2017;144(4):624–634. 28087628 10.1242/dev.146928 PMC5312040

[8] Chan AL, La HM, Legrand JMD, Mäkelä JA, Eichenlaub M, De Seram M, Ramialison M, Hobbs RM. Germline stem cell activity is sustained by SALL4-dependent silencing of distinct tumor suppressor genes. Stem Cell Rep. 2017;9(3):956–971. 10.1016/j.stemcr.2017.08.001 PMC5599261 28867346

[9] Lee SJ, Park J, Lee DJ, Otsu K, Kim P, Mizuno S, Lee MJ, Kim HY, Harada H, Takahashi S, et al. *Mast4* knockout shows the regulation of spermatogonial stem cell self-renewal via the FGF2/ERM pathway. Cell Death Differ. 2021;28(5):1441–1454. 33219327 10.1038/s41418-020-00670-2 PMC8167111

[10] Codino A, Turowski T, van de Lagemaat LN, Ivanova I, Tavosanis A, Much C, Auchynnikava T, Vasiliauskaitė L, Morgan M, Rappsilber J, et al. NANOS2 is a sequence-specific mRNA-binding protein that promotes transcript degradation in spermatogonial stem cells. iScience. 2021;24(7): Article 102762. 34278268 10.1016/j.isci.2021.102762 PMC8271163

[11] Fan S, Jiao Y, Khan R, Jiang X, Javed AR, Ali A, Zhang H, Zhou J, Naeem M, Murtaza G, et al. Homozygous mutations in C14orf39/SIX6OS1 cause non-obstructive azoospermia and premature ovarian insufficiency in humans. Am J Hum Genet. 2021;108(2):324–336. 33508233 10.1016/j.ajhg.2021.01.010 PMC7895996

[12] Liu C, Tu C, Wang L, Wu H, Houston BJ, Mastrorosa FK, Zhang W, Shen Y, Wang J, Tian S, et al. Deleterious variants in X-linked CFAP47 induce asthenoteratozoospermia and primary male infertility. Am J Hum Genet. 2021;108(2):309–323. 33472045 10.1016/j.ajhg.2021.01.002 PMC7895902

[13] Wang F, Wang L, Gan S, Feng S, Ouyang S, Wang X, Yuan S. SERBP1 promotes stress granule clearance by regulating 26S proteasome activity and G3BP1 ubiquitination and protects male germ cells from thermostimuli damage. Research. 2023;6:0091. 37223481 10.34133/research.0091 PMC10202183

[14] Oud MS, Volozonoka L, Smits RM, Vissers L, Ramos L, Veltman JA. A systematic review and standardized clinical validity assessment of male infertility genes. Hum Reprod. 2019;34(5):932–941. 30865283 10.1093/humrep/dez022 PMC6505449

[15] Tu C, Cong J, Zhang Q, He X, Zheng R, Yang X, Gao Y, Wu H, Lv M, Gu Y, et al. Bi-allelic mutations of DNAH10 cause primary male infertility with asthenoteratozoospermia in humans and mice. Am J Hum Genet. 2021;108(8):1466–1477. 34237282 10.1016/j.ajhg.2021.06.010 PMC8387467

[16] Chen M, Yao C, Qin Y, Cui X, Li P, Ji Z, Lin L, Wu H, Zhou Z, Gui Y, et al. Mutations of MSH5 in nonobstructive azoospermia (NOA) and rescued via in vivo gene editing. Signal Transduct Target Ther. 2022;7(1):1. 34980881 10.1038/s41392-021-00710-4 PMC8724278

[17] Tan YQ, Tu C, Meng L, Yuan S, Sjaarda C, Luo A, Du J, Li W, Gong F, Zhong C, et al. Loss-of-function mutations in TDRD7 lead to a rare novel syndrome combining congenital cataract and nonobstructive azoospermia in humans. Genet Med. 2019;21(5):1209–1217. 31048812 10.1038/gim.2017.130

[18] Cui Y, Chen W, Du L, He Z. OIP5 interacts with NCK2 to mediate human spermatogonial stem cell self-renewal and apoptosis through cell cyclins and cycle progression and its abnormality is correlated with male infertility. Research. 2023;6:0162. 37292517 10.34133/research.0162 PMC10246317

[19] Fu H, Zhang W, Yuan Q, Niu M, Zhou F, Qiu Q, Mao G, Wang H, Wen L, Sun M, et al. PAK1 promotes the proliferation and inhibits apoptosis of human spermatogonial stem cells via PDK1/KDR/ZNF367 and ERK1/2 and AKT pathways. Mol Ther Nucleic Acids. 2018;12:769–786. 30141410 10.1016/j.omtn.2018.06.006 PMC6111072

[20] Du L, Chen W, Li C, Cui Y, He Z. RNF144B stimulates the proliferation and inhibits the apoptosis of human spermatogonial stem cells via the FCER2/NOTCH2/HES1 pathway and its abnormality is associated with azoospermia. J Cell Physiol. 2022;237(9):3565–3577. 35699595 10.1002/jcp.30813

[21] Gao J, Xu Z, Song W, Huang J, Liu W, He Z, He L. USP11 regulates proliferation and apoptosis of human spermatogonial stem cells via HOXC5-mediated canonical WNT/β-catenin signaling pathway. Cell Mol Life Sci. 2024;81(1):211. 38722330 10.1007/s00018-024-05248-6 PMC11082041

[22] Chen W, Cui Y, Li C, He C, Du L, Liu W, He Z. KLF2 controls proliferation and apoptosis of human spermatogonial stem cells via targeting GJA1. iScience. 2024;27(2): Article 109024. 38352225 10.1016/j.isci.2024.109024 PMC10863320

[23] Yu FX, Zhao B, Guan KL. Hippo pathway in organ size control, tissue homeostasis, and cancer. Cell. 2015;163(4):811–828. 26544935 10.1016/j.cell.2015.10.044 PMC4638384

[24] Pan D. The hippo signaling pathway in development and cancer. Dev Cell. 2010;19(4):491–505. 20951342 10.1016/j.devcel.2010.09.011 PMC3124840

[25] Shreberk-Shaked M, Oren M. New insights into YAP/TAZ nucleo-cytoplasmic shuttling: New cancer therapeutic opportunities? Mol Oncol. 2019;13(6):1335–1341. 31050214 10.1002/1878-0261.12498 PMC6547617

[26] Tsoi M, Morin M, Rico C, Johnson RL, Paquet M, Gévry N, Boerboom D. *Lats1* and *Lats2* are required for ovarian granulosa cell fate maintenance. FASEB J. 2019;33(10):10819–10832. 31268774 10.1096/fj.201900609R PMC6766663

[27] Kawamura K, Cheng Y, Suzuki N, Deguchi M, Sato Y, Takae S, Ho CH, Kawamura N, Tamura M, Hashimoto S, et al. Hippo signaling disruption and Akt stimulation of ovarian follicles for infertility treatment. Proc Natl Acad Sci USA. 2013;110(43):17474–17479. 24082083 10.1073/pnas.1312830110 PMC3808580

[28] Meng C, Tian G, Xu C, Li X, Zhang Y, Wang Y, Qin J, Fok EKL, Hinton BT, Mak KK, et al. Hippo kinases MST1 and MST2 control the differentiation of the epididymal initial segment via the MEK-ERK pathway. Cell Death Differ. 2020;27(10):2797–2809. 32332916 10.1038/s41418-020-0544-x PMC7492226

[29] Yin Q, Liu C, Jiang WY, Gong HH, Li CY, He ZP. The roles and regulation of Yes-associated protein 1 in stem cells. Biocell. 2023;47(1):33–39.

[30] Lv X, He C, Huang C, Wang H, Hua G, Wang Z, Zhou J, Chen X, Ma B, Timm BK, et al. Timely expression and activation of YAP1 in granulosa cells is essential for ovarian follicle development. FASEB J. 2019;33(9):10049–10064. 31199671 10.1096/fj.201900179RR PMC6704445

[31] Oh SH, Swiderska-Syn M, Jewell ML, Premont RT, Diehl AM. Liver regeneration requires Yap1-TGFβ-dependent epithelial-mesenchymal transition in hepatocytes. J Hepatol. 2018;69(2):359–367. 29758331 10.1016/j.jhep.2018.05.008 PMC6349217

[32] Schlegelmilch K, Mohseni M, Kirak O, Pruszak J, Rodriguez JR, Zhou D, Kreger BT, Vasioukhin V, Avruch J, Brummelkamp TR, et al. Yap1 acts downstream of α-catenin to control epidermal proliferation. Cell. 2011;144(5):782–795. 21376238 10.1016/j.cell.2011.02.031 PMC3237196

[33] LeBlanc L, Ramirez N, Kim J. Context-dependent roles of YAP/TAZ in stem cell fates and cancer. Cell Mol Life Sci. 2021;78(9):4201–4219. 33582842 10.1007/s00018-021-03781-2 PMC8164607

[34] Kaneshige A, Kaji T, Zhang L, Saito H, Nakamura A, Kurosawa T, Ikemoto-Uezumi M, Tsujikawa K, Seno S, Hori M, et al. Relayed signaling between mesenchymal progenitors and muscle stem cells ensures adaptive stem cell response to increased mechanical load. Cell Stem Cell. 2022;29(2):265–280.e6. 34856120 10.1016/j.stem.2021.11.003

[35] Kim JY, Park R, Lee JH, Shin J, Nickas J, Kim S, Cho SH. Yap is essential for retinal progenitor cell cycle progression and RPE cell fate acquisition in the developing mouse eye. Dev Biol. 2016;419(2):336–347. 27616714 10.1016/j.ydbio.2016.09.001 PMC5125893

[36] Lavado A, Park JY, Paré J, Finkelstein D, Pan H, Xu B, Fan Y, Kumar RP, Neale G, Kwak YD, et al. The Hippo pathway prevents YAP/TAZ-driven hypertranscription and controls neural progenitor number. Dev Cell. 2018;47(5):576–591.e8. 30523785 10.1016/j.devcel.2018.09.021 PMC6296252

[37] Liu Z, Lin L, Zhu H, Wu Z, Ding X, Hu R, Jiang Y, Tang C, Ding S, Guo R. YAP promotes cell proliferation and stemness maintenance of porcine muscle stem cells under high-density condition. Cells. 2021;10(11):3069. 34831292 10.3390/cells10113069 PMC8621012

[38] Wang Z, Cui M, Qu Y, He R, Wu W, Lin H, Shao Z. Hypoxia protects rat bone marrow mesenchymal stem cells against compression-induced apoptosis in the degenerative disc microenvironment through activation of the HIF-1α/YAP signaling pathway. Stem Cells Dev. 2020;29(20):1309–1319. 32799744 10.1089/scd.2020.0061

[39] Chen X, Li Y, Luo J, Hou N. Molecular mechanism of Hippo-YAP1/TAZ pathway in heart development, disease, and regeneration. Front Physiol. 2020;11:389. 32390875 10.3389/fphys.2020.00389 PMC7191303

[40] Onn I, Heidinger-Pauli JM, Guacci V, Unal E, Koshland DE. Sister chromatid cohesion: A simple concept with a complex reality. Annu Rev Cell Dev Biol. 2008;24:105–129. 18616427 10.1146/annurev.cellbio.24.110707.175350

[41] Peters JM, Tedeschi A, Schmitz J. The cohesin complex and its roles in chromosome biology. Genes Dev. 2008;22(22):3089–3114. 19056890 10.1101/gad.1724308

[42] Nasmyth K, Haering CH. Cohesin: Its roles and mechanisms. Annu Rev Genet. 2009;43:525–558. 19886810 10.1146/annurev-genet-102108-134233

[43] Kagey MH, Newman JJ, Bilodeau S, Zhan Y, Orlando DA, van Berkum NL, Ebmeier CC, Goossens J, Rahl PB, Levine SS, et al. Mediator and cohesin connect gene expression and chromatin architecture. Nature 2010;467(7314):430–435. 20720539 10.1038/nature09380 PMC2953795

[44] Biswas U, Hempel K, Llano E, Pendas A, Jessberger R. Distinct roles of meiosis-specific cohesin complexes in mammalian spermatogenesis. PLOS Genet. 2016;12(10): Article e1006389. 27792785 10.1371/journal.pgen.1006389 PMC5085059

[45] Yan J, Enge M, Whitington T, Dave K, Liu J, Sur I, Schmierer B, Jolma A, Kivioja T, Taipale M, et al. Transcription factor binding in human cells occurs in dense clusters formed around cohesin anchor sites. Cell. 2013;154(4):801–813. 23953112 10.1016/j.cell.2013.07.034

[46] Fisher JB, Peterson J, Reimer M, Stelloh C, Pulakanti K, Gerbec ZJ, Abel AM, Strouse JM, Strouse C, McNulty M, et al. The cohesin subunit Rad21 is a negative regulator of hematopoietic self-renewal through epigenetic repression of Hoxa7 and Hoxa9. Leukemia. 2017;31(3):712–719. 27554164 10.1038/leu.2016.240 PMC5332284

[47] Zhang Q, Chen HJ, Xie CZ, Qiu GF. Potential role for the germ cell-specific *Rad21* in early meiosis of oocyte and spermatocyte in the Chinese mitten crab *Eriocheir sinensis*. Gene. 2023;862:147262. 36764338 10.1016/j.gene.2023.147262

[48] van der Bijl N, Röpke A, Biswas U, Wöste M, Jessberger R, Kliesch S, Friedrich C, Tüttelmann F. Mutations in the stromal antigen 3 (STAG3) gene cause male infertility due to meiotic arrest. Hum Reprod. 2019;34(11):2112–2119. 31682730 10.1093/humrep/dez204

[49] Xu H, Beasley MD, Warren WD, van der Horst GT, McKay MJ. Absence of mouse REC8 cohesin promotes synapsis of sister chromatids in meiosis. Dev Cell. 2005;8(6):949–961. 15935783 10.1016/j.devcel.2005.03.018

[50] Deng P, Wang Z, Chen J, Liu S, Yao X, Liu S, Liu L, Yu Z, Huang Y, Xiong Z, et al. RAD21 amplification epigenetically suppresses interferon signaling to promote immune evasion in ovarian cancer. J Clin Invest. 2022;132(22):e159628. 36201246 10.1172/JCI159628 PMC9663158

[51] Yu C, Ji SY, Dang YJ, Sha QQ, Yuan YF, Zhou JJ, Yan LY, Qiao J, Tang F, Fan HY. Oocyte-expressed yes-associated protein is a key activator of the early zygotic genome in mouse. Cell Res. 2016;26(3):275–287. 26902285 10.1038/cr.2016.20 PMC4783469

[52] Sha QQ, Zhu YZ, Li S, Jiang Y, Chen L, Sun XH, Shen L, Ou XH, Fan HY. Characterization of zygotic genome activation-dependent maternal mRNA clearance in mouse. Nucleic Acids Res. 2020;48(2):879–894. 31777931 10.1093/nar/gkz1111 PMC6954448

[53] Chen W, Cui Y, Liu B, Li C, Du L, Tang R, Qin L, Jiang Y, Li J, Yu X, et al. Hsa-miR-1908-3p mediates the self-renewal and apoptosis of human spermatogonial stem cells via targeting KLF2. Mol Ther Nucleic Acids. 2020;20:788–800. 32438314 10.1016/j.omtn.2020.04.016 PMC7240205

