# Supplementary material for: Essential Regulation of YAP1 in Fate Determinations of Spermatogonial Stem Cells and Male Fertility by Interacting with RAD21 and Targeting NEDD4 in Humans and Mice
Source: Research (Wash D C). 2024 Dec 10;7:0544. doi: 10.34133/research.0544 (PMC11628678; doi:10.34133/research.0544)
Supplement: Supplementary 1 — Figs. S1 to S9 Tables S1 to S7 [file research.0544.f1.docx]

**Essential regulation of YAP1 in fate determinations of spermatogonial stem cells and male fertility by interacting with RAD21 and targeting NEDD4 in humans and mice**

Chunyun Li^1^, Wei Chen^1^, Yinghong Cui^1^, Dong Zhang^1^, Qingqing Yuan^3^, Xing Yu^1^, Zuping He^1,2*^

**Supplementary Data**

**Supplemental Figures 1-10**

**Fig. S1**. The regulation and interaction of PDK1 and YAP1 in human SSCs.

**Fig. S2.** Influence of *YAP1* P45L mutations on proliferation and apoptosis of human SSCs.

**Fig. S3.** The identification of human SSC line.

**Figure S4.** The effect of YAP1 siRNA on human SSC line in vivo.

**Figure S5.** The electropherograms of RNA sequencing.

**Figure S6.** Evaluation of NEDD4 knockdown efficiency.

**Figure S7.** Construction of NEDD4 overexpression plasmid.

**Figure S8.** Assessment of RAD21 knockdown efficiency.

**Figure S9.** Genotype identification of *Yap1* cKO mice.

**~~Figure S10.~~** ~~The functions and mechanisms of YAP1 and its dysfunction in mediating fate determinations of SSCs and spermatogenesis.~~

**Supplemental Tables 1-7**

**Table S1**. Identification of *YAP1* single nucleotide variants in whole exome sequencing from (WES) 777 NOA patients

**Table S2**. Pathogenicity prediction of missense variant sites in *YAP1* gene

**Table S3**. The prediction of RAD21 banding sites in NEDDD4 promoter using hTFtarget

**Table S4**. Gene primers used for RT-PCR and real time RT-PCR.

**Table S5**. The detailed information of antibodies for immunocytochemistry and immunohistochemistry.

**Table S6**. The siRNA sequences for PDK1, YAP1, NEDD4, and RAD21 oligonucleotides.

**Table S7**. The detailed information of antibodies for Western blots and immunoprecipitation (IP).

**
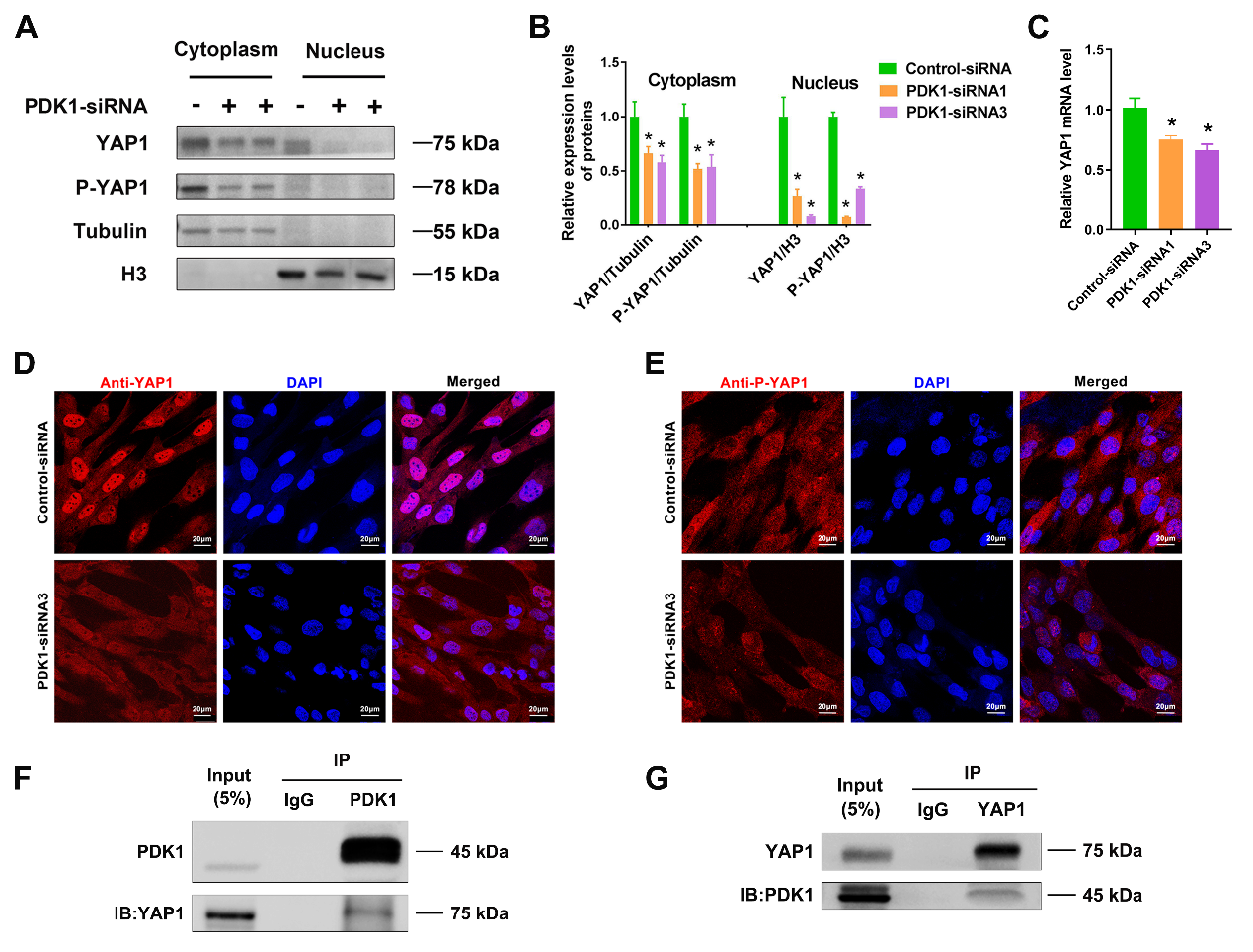
**

**Figure S1. The regulation and interaction of PDK1 and YAP1 in human SSCs.** (A and B) Western blots showed that expression changes of YAP1 and phos-YAP1 proteins in the cytoplasm and nuclei of human SSCs treated by PDK1-siRNA1 and PDK1-siRNA3. Tubulin and H3 proteins were used as the controls of loading proteins. **p*<0.05. (C) Real-time PCR indicated that transcripts of *YAP1* in human SSCs treated with PDK1-siRNA3 and control-siRNA. **p*<0.05. (D and E) Immunocytochemistry displayed that protein expression of YAP1 (D) and phos-YAP1 (E) in human SSCs after treatment of PDK1-siRNA3 and control-siRNA.

**
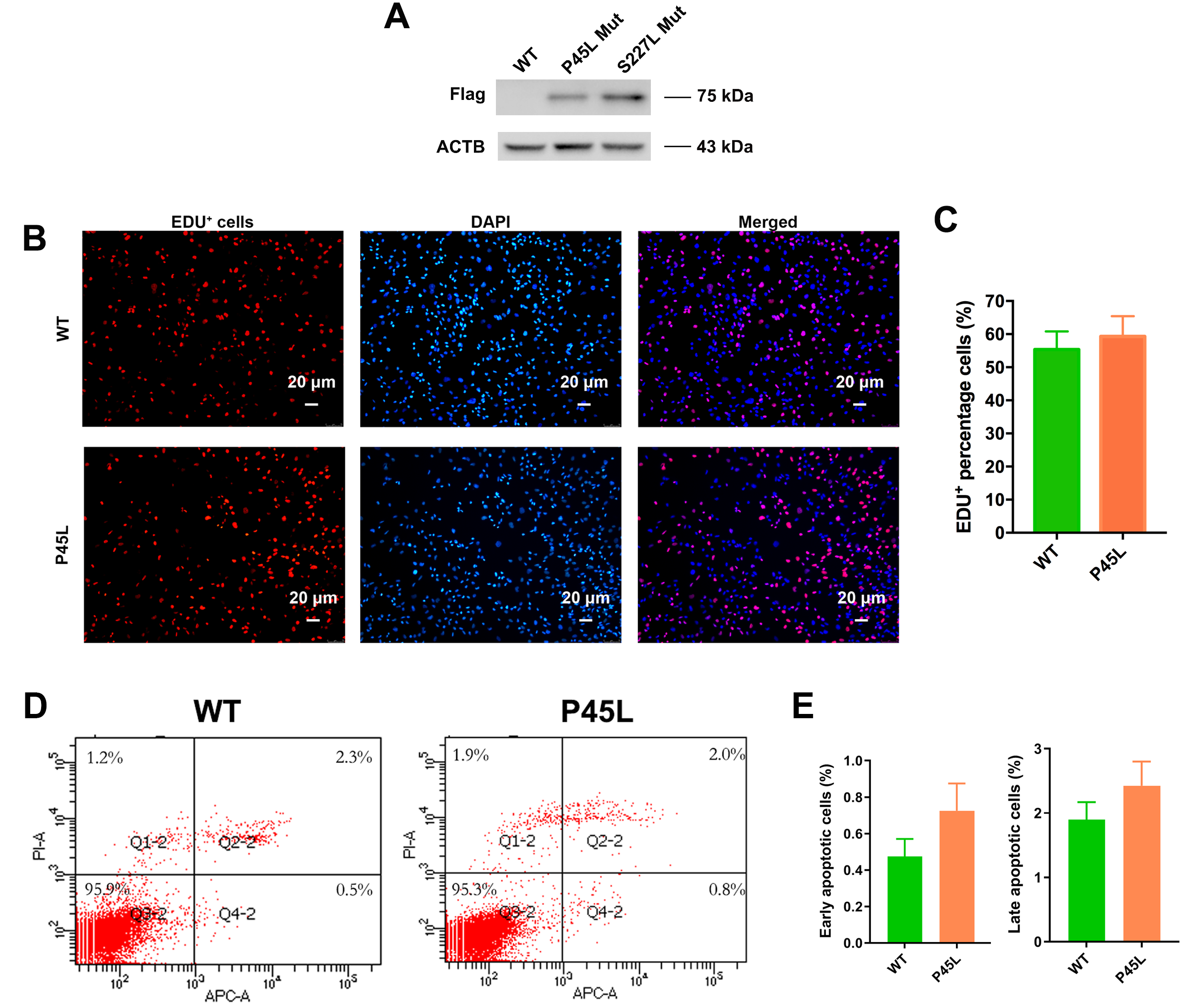
**

**Figure S2. Influence of *YAP1* mutations on the proliferation and apoptosis of human SSCs.** (A) Western blots demonstrated the construction of *YAP1* mutations of S227L and P45L and with anti-flag. (B-C) (G-H) EDU incorporation assay displayed DNA replication of human SSCs with the P45L mutation of *YAP1* and WT. (I-J) Annexin V-APC/PI and flow cytometry analysis demonstrated apoptosis of human SSCs treated with P45L mutation and WT of *YAP1*.

**
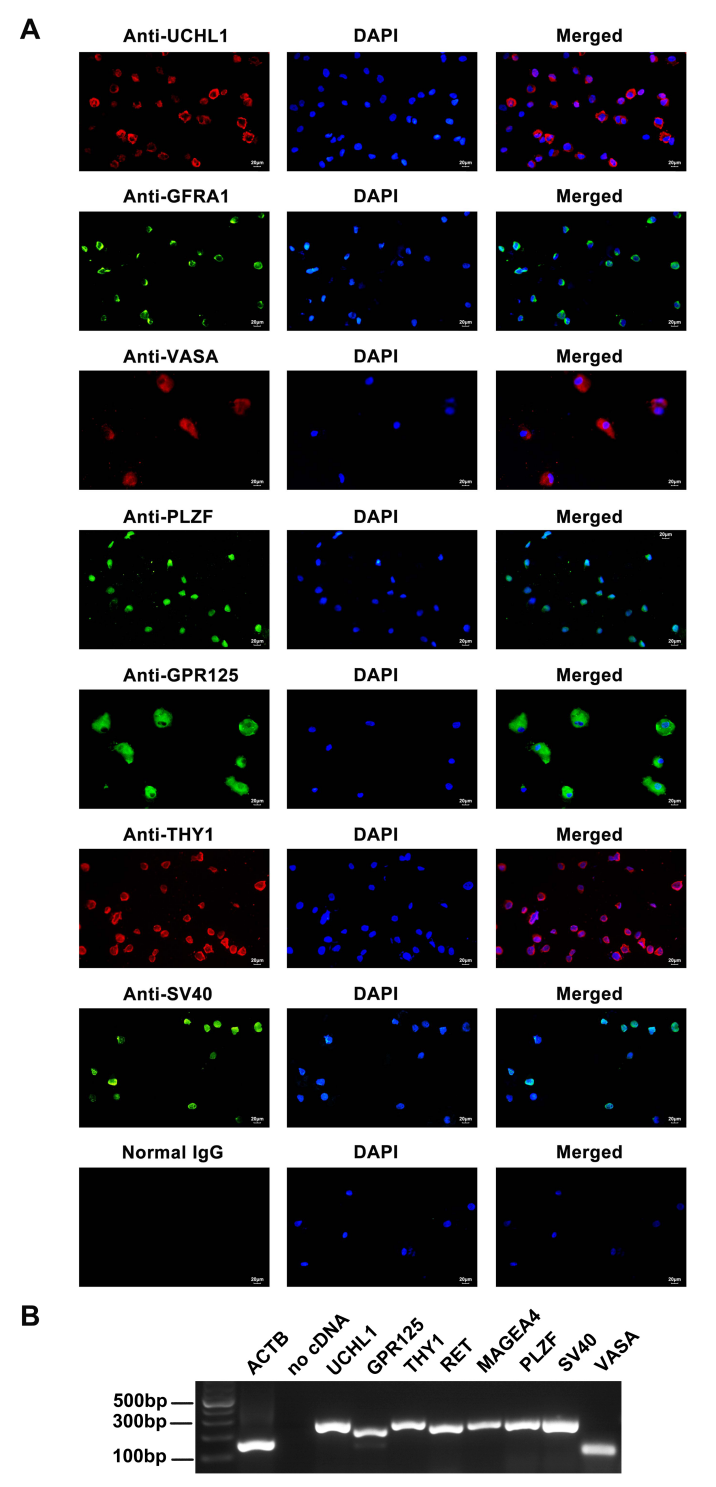
**

**Figure S3. The phenotypic identification of human SSC line.** (A) Immunocytochemistry showed the expression of UCHL1, GFRA1, VASA, PLZF, GPR125, THY1, and SV40 in human SSC line. Normal IgG was used as a negative control. Scale bars: 20 μm. (B) RT-PCR revealed that *UCHL1*, *GPR125*, *THY1*, *RET*, *MAGEA4*, *PLZF*, S*V40*, and *VASA* were expressed in human SSC line. *ACTB* and no cDNA were utilized as a positive control and a negative control, respectively.


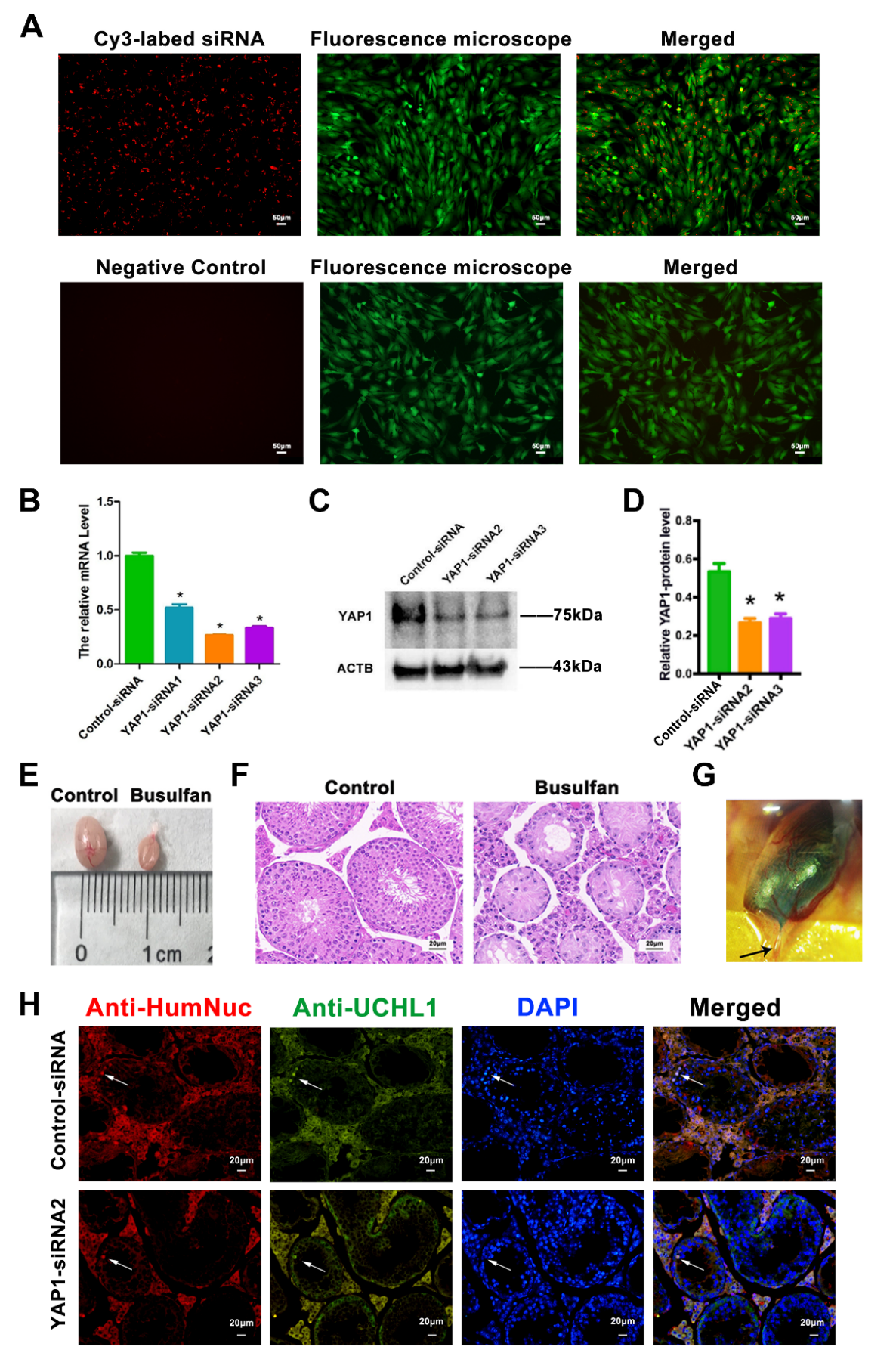


**Figure S4. The effect of YAP1 siRNA on human SSC line *in vivo* and *vitro*.** (A) Fluorescence microscope displayed transfection efficiency of Cy3-labeled siRNA into human SSCs. Scale bars: 50 μm. (B) Real-time PCR revealed mRNA of *YAP1* in human SSCs transfected with YAP1-siRNA1, 2 and 3. (C-D) Western blots showed the expression of YAP1 protein in human SSCs transfected with YAP1-siRNA2 and YAP1-siRNA3. ACTB served as a control of loading proteins. (E) The testes derived from mice treated with busulfan and without busulfan (control). (F) H&E staining displayed testis tissues of the control and busulfan-treated group. (G) The testes of mice were transplanted with human SSCs. The black arrow indicated the efferent duct of testes. (H) Double immunostaining illustrated co-expression of human nuclear antigen (HumNuc) and UCHL1 (arrows indicated the positive cells) in seminiferous tubules of the recipient mice transplanted with human SSCs with control-siRNA and YAP1-siRNA2 transfection.


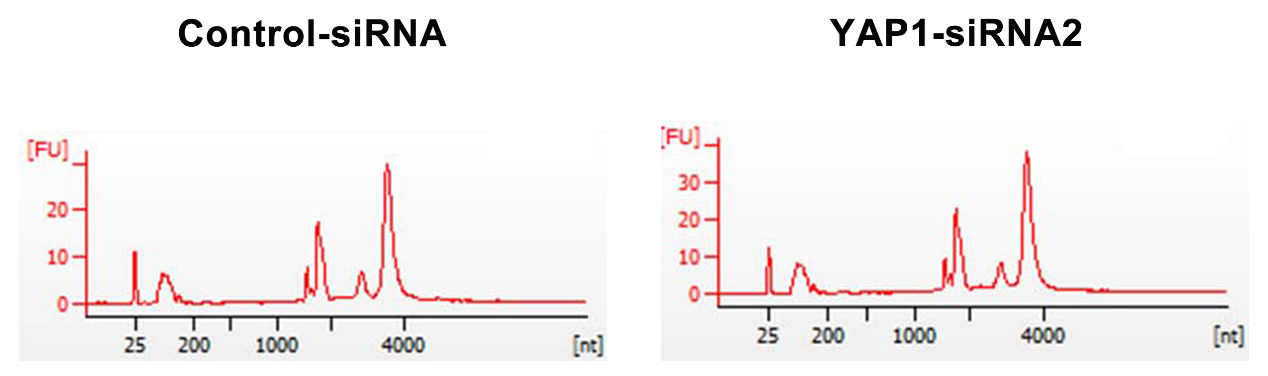


**Figure S5. The electropherograms of total RNA for RNA sequencing.** Agilent Bioanalyzer captured the electropherograms RNAs of control-siRNA group (A) and YAP1-siRNA2 (B) for RNA sequencing.

**
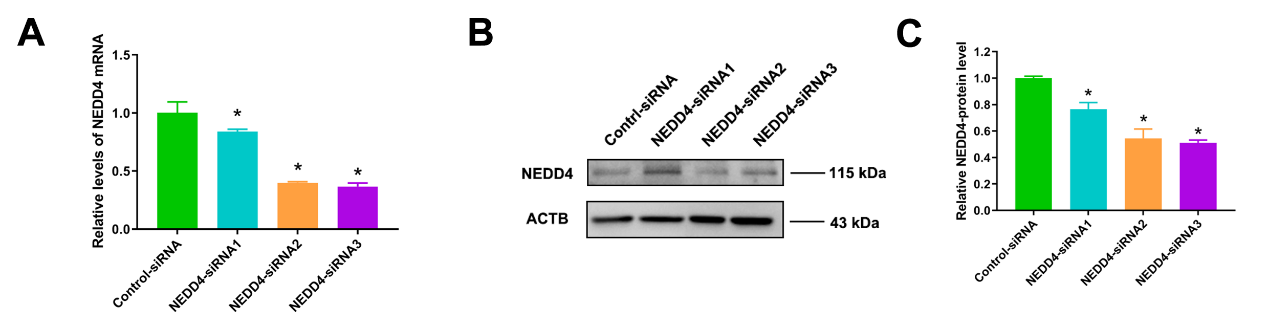
**

**Figure S6.** **Evaluation of NEDD4 knockdown efficiency.** (A) Real-time PCR displayed the mRNA changes of *NEDD4* in human SSCs after transfection with NEDD4-siRNA1, NEDD4-siRNA2, and NEDD4-siRNA3. (B-C) Western blots indicated NEDD4 protein expression of human SSCs transfected with NEDD4-siRNA1, NEDD4-siRNA2, and NEDD4-siRNA3.

**
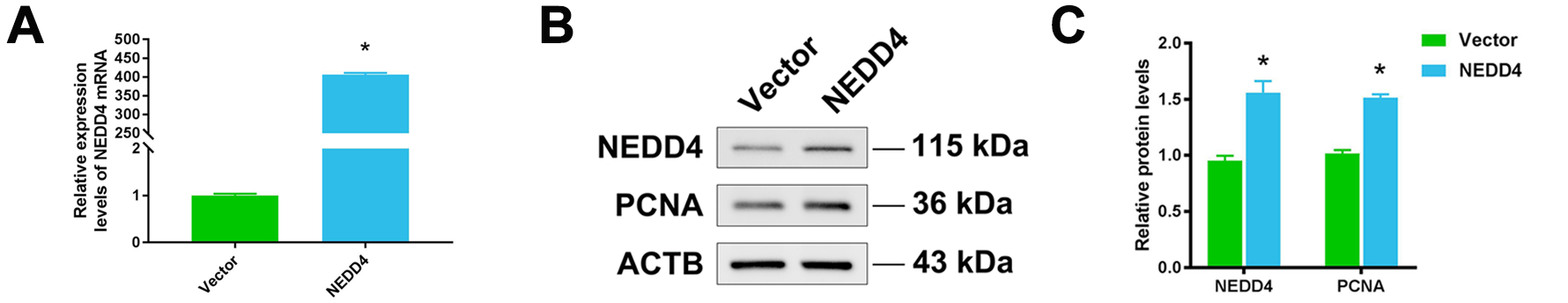
**

**Figure S7. Construction of NEDD4 overexpression plasmid.** (A-C) Real-time PCR and Western blots revealed *NEDD4* mRNA level (A), NEDD4 (B and C), and PCNA (B and C) protein expression of human SSCs treated with NEDD4 plasmid and vector.

**
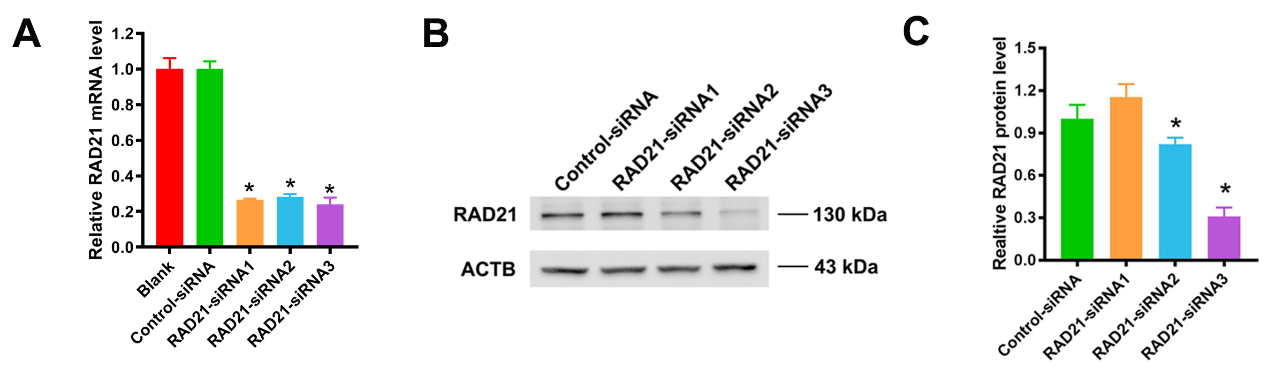
**

**Figure S8. Assessment of RAD21 knockdown efficiency.** (A-C) Real-time PCR and Western blots demonstrated the effect of RAD21-siRNAs on *RAD21* mRNA (A) and RDA21 protein expression (B and C) in human SSCs.

**Figure S9**

**
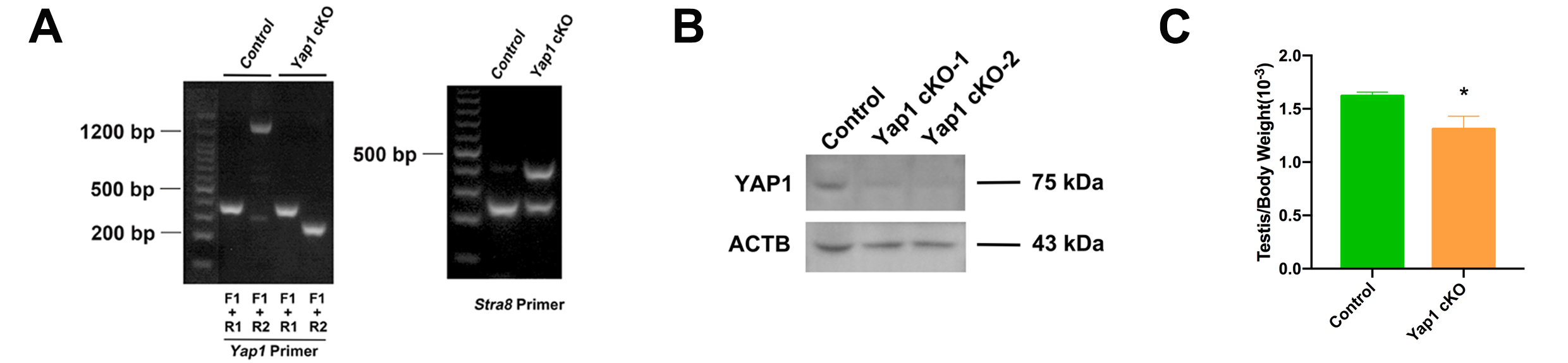
**

**Figure S9. Genotype identification of *Yap1* cKO mice.** (A) RT-PCR identified the genotype of *Yap1* cKO mice. (B) Western blots of Yap1 expression in control wild type mice and *Yap1* cKO mice. (C) The ration of testicular body and weight in *Yap1* cKO mice and control wild type mice.

**Supplemental Tables 1-7**

**Table S1. Identification of *YAP1* single nucleotide variants (SNVs) by WES from 777 NOA patients**

| **Variants locations** | **Nucleotide changes** | **Amino acid**  **changes** | **Heterozygote/**  **homozygous** | **SNPs（rs#）** |
| --- | --- | --- | --- | --- |
| exon 1 | c.42G>A^*^ | p.Q14Q | 1/0 | rs1942851760 |
| exon 1 | c.55C>A^*#^ | p.P19T | 1/0 | - |
| exon 1 | c.57G>A | p.P19P | 0/1 | rs937455182 |
| exon 1 | c.134C>T^*#^ | p.P45L | 1/0 | rs1365255865 |
| exon 2 | c.387A>G | p.P129P | 1/0 | rs773991529^△^ |
| exon 2 | c.567C>T | p.F189F | 1/0 | rs757399180^△^ |
| exon 3 | c.680C>T^#^ | p.S227L | 1/0 | rs376161041 |
| exon 5 | c.879C>T | p.G293G | 1/0 | rs756175059^△^ |
| exon 6 | c.1016C>T^#^ | p.P339L | 3/0 | rs527326391^△^ |
| exon 7 | c.1116C>G^*^ | p.P372P | 1/0 | - |
| exon 9 | c.1482G>A^*^ | p.L494L | 1/0 | rs1591486907 |
| intron 3 | c.689-10T>C^*^ | - | 3/0 | - |

**Notes**: * indicated a novel variation; ^#^ denoted probably damaging; ^△^ indicated the abnormality of allele increases risk of NOA.

**Table S2. Pathogenicity prediction of missense variant sites in *YAP1* gene**

| **Nucleotide changes** | **Amino acid changes** | **SNP No.** | **SIFT**  **(scores)** | **Polyphen-2 HDIV(scores)** | **MutationTaster** | **M-Cap** |
| --- | --- | --- | --- | --- | --- | --- |
| c.55C>A | p.P19T | -- | Deleterious  (0) | Benign  (0) | Polymorphism | Tolerable |
| c.134C>T | p.P45L | rs1365255865 | Deleterious (0.04) | Benign  (0) | Polymorphism | Tolerable |
| c.680C>T | p.S227L | rs376161041 | Tolerable  (0.414) | Probably damaging  (0.959) | Disease causing | Damaging |
| c.1016C>T | p.P339L | rs527326391 | Deleterious (0.006) | Benign  (0.447) | Disease causing | Damaging |

**Table S3. The prediction of RAD21 banding sites in NEDDD4 promoter using hTFtarget**

TF **Pattern name Source Sequence name Srart Stop Score P value Q value Matched motif**

RAD21 m-dataset-2628-1 hTFtarget NC_000015.10:c55995612-55993612 1239 1254 + 18.5263 3.74e-07 0.00143 ACTGCCTTCTGGTGGT

RAD21 m-dataset-2608-1 hTFtarget NC_000015.10:c55995612-55993612 1238 1253 + 18.0455 4.83e-07 0.00187 CACTGCCTTCTGGTGG

RAD21 m-dataset-2597-1 hTFtarget NC_000015.10:c55995612-55993612 1238 1253 + 17.7576 6.23e-07 0.00244 CACTGCCTTCTGGTGG

RAD21 m-dataset-2616-1 hTFtarget NC_000015.10:c55995612-55993612 1235 1254 + 17.5 6.49e-07 0.00247 CGTCACTGCCTTCTGGTGGT

RAD21 m-dataset-2601-1 hTFtarget NC_000015.10:c55995612-55993612 1238 1253 + 17.4394 7.32e-07 0.00281 CACTGCCTTCTGGTGG

RAD21 m-dataset-2607-1 hTFtarget NC_000015.10:c55995612-55993612 1238 1253 + 16.9545 1.01e-06 0.00393 CACTGCCTTCTGGTGG

RAD21 m-dataset-2614-1 hTFtarget NC_000015.10:c55995612-55993612 1235 1255 + 16.7273 1.01e-06 0.00387 CGTCACTGCCTTCTGGTGGTG

RAD21 m-dataset-2612-1 hTFtarget NC_000015.10:c55995612-55993612 1238 1253 + 16.8788 1.07e-06 0.00418 CACTGCCTTCTGGTGG

RAD21 m-dataset-2599-1 hTFtarget NC_000015.10:c55995612-55993612 1238 1253 + 16.5455 1.31e-06 0.00513 CACTGCCTTCTGGTGG

RAD21 m-dataset-2623-1 hTFtarget NC_000015.10:c55995612-55993612 1232 1253 + 16.0857 1.65e-06 0.00639 CTTCGTCACTGCCTTCTGGTGG

RAD21 m-dataset-2629-1 hTFtarget NC_000015.10:c55995612-55993612 1238 1253 + 15.9143 2.04e-06 0.00778 CACTGCCTTCTGGTGG

RAD21 m-dataset-2617-1 hTFtarget NC_000015.10:c55995612-55993612 1239 1256 + 15.197 3.01e-06 0.0115 ACTGCCTTCTGGTGGTGC

RAD21 m-dataset-2624-1 hTFtarget NC_000015.10:c55995612-55993612 1232 1253 + 13.8286 4.25e-06 0.0161 CTTCGTCACTGCCTTCTGGTGG

RAD21 m-dataset-2615-1 hTFtarget NC_000015.10:c55995612-55993612 1241 1256 + 14.7727 4.37e-06 0.0167 TGCCTTCTGGTGGTGC

RAD21 m-dataset-2622-1 hTFtarget NC_000015.10:c55995612-55993612 1232 1253 + 13.9571 4.51e-06 0.0167 CTTCGTCACTGCCTTCTGGTGG

RAD21 m-dataset-2605-1 hTFtarget NC_000015.10:c55995612-55993612 1240 1252 + 14.6818 5.36e-06 0.0208 CTGCCTTCTGGTG

RAD21 m-dataset-2605-1 hTFtarget NC_000015.10:c55995612-55993612 1969 1981 + 10.6667 4.96e-05 0.0962 CCGCCTCCTCCCG

RAD21 m-dataset-2614-1 hTFtarget NC_000015.10:c55995612-55993612 1828 1848 + 8.12121 7.61e-05 0.12 CACGCGCGCCCCCGCCCAGCC

RAD21 m-dataset-2624-1 hTFtarget NC_000015.10:c55995612-55993612 1455 1476 + 6.31429 8.65e-05 0.164 ACGCTCAGGCTTTCCCTGGCGG

RAD21 m-dataset-2614-1 hTFtarget NC_000015.10:c55995612-55993612 1419 1439 + 7.60606 9.4e-05 0.12 CTGCTGCGATCCCTCCAGGCC

RAD21 m-dataset-2603-1 hTFtarget NC_000015.10:c55995612-55993612 1239 1254 - 18.0303 4.74e-07 0.00185 ACCACCAGAAGGCAGT

RAD21 m-dataset-2626-1 hTFtarget NC_000015.10:c55995612-55993612 1238 1253 - 17.7429 6.16e-07 0.0024 CCACCAGAAGGCAGTG

RAD21 m-dataset-2613-1 hTFtarget NC_000015.10:c55995612-55993612 1238 1253 - 17.4242 7.64e-07 0.00297 CCACCAGAAGGCAGTG

RAD21 m-dataset-2610-1 hTFtarget NC_000015.10:c55995612-55993612 1238 1253 - 17.3182 7.86e-07 0.00304 CCACCAGAAGGCAGTG

RAD21 m-dataset-2618-1 hTFtarget NC_000015.10:c55995612-55993612 1239 1254 - 17.2727 8.03e-07 0.00312 ACCACCAGAAGGCAGT

RAD21 m-dataset-2602-1 hTFtarget NC_000015.10:c55995612-55993612 1238 1253 - 17.2121 8.85e-07 0.00346 CCACCAGAAGGCAGTG

RAD21 m-dataset-2606-1 hTFtarget NC_000015.10:c55995612-55993612 1238 1253 - 17.1364 9e-07 0.00345 CCACCAGAAGGCAGTG

RAD21 m-dataset-2609-1 hTFtarget NC_000015.10:c55995612-55993612 1238 1253 - 17.0758 9.58e-07 0.00378 CCACCAGAAGGCAGTG

RAD21 m-dataset-2611-1 hTFtarget NC_000015.10:c55995612-55993612 1238 1253 - 16.8788 1.1e-06 0.00431 CCACCAGAAGGCAGTG

RAD21 m-dataset-2598-1 hTFtarget NC_000015.10:c55995612-55993612 1239 1254 - 16.7576 1.16e-06 0.00452 ACCACCAGAAGGCAGT

RAD21 m-dataset-2619-1 hTFtarget NC_000015.10:c55995612-55993612 1242 1253 - 15.803 2.35e-06 0.00917 CCACCAGAAGGC

RAD21 m-dataset-2625-1 hTFtarget NC_000015.10:c55995612-55993612 1237 1252 - 15.1842 3.22e-06 0.0124 CACCAGAAGGCAGTGA

RAD21 m-dataset-2600-1 hTFtarget NC_000015.10:c55995612-55993612 1239 1252 - 15.4091 3.29e-06 0.0129 CACCAGAAGGCAGT

RAD21 m-dataset-2604-1 hTFtarget NC_000015.10:c55995612-55993612 1242 1254 - 13.6515 1.09e-05 0.0427 ACCACCAGAAGGC

RAD21 m-dataset-2622-1 hTFtarget NC_000015.10:c55995612-55993612 1849 1870 - 11.0429 1.88e-05 0.0348 GGCCGGCGGCGCGCGCTCCTGG

RAD21 m-dataset-2623-1 hTFtarget NC_000015.10:c55995612-55993612 1849 1870 - 9.68571 5.08e-05 0.0982 GGCCGGCGGCGCGCGCTCCTGG

RAD21 m-dataset-2625-1 hTFtarget NC_000015.10:c55995612-55993612 1966 1981 - 8.89474 5.46e-05 0.105 CGGGAGGAGGCGGGGA

**Table S4. Gene primers used for RT-PCR and real-time PCR**

| **Genes** | **Species** | **Forward primers (5'-3')** | **Reverse primers (5'-3')** | **Product**  **sizes** |
| --- | --- | --- | --- | --- |
| *ACTB* | Human | CCTGGCACCCAGCACAAT | GGGCCGGACTCGTCATAC | 144 |
| *YAP1* | Human | TAGCCCTGCGTAGCCAGTTA | TCATGCTTAGTCCACTGTCTGT | 177 |
| *NEEDD4* | Human | TCCAATGATCTAGGGCCTTTACC | TCCAACCGAGGATCTTCCCAT | 116 |
| *RAD21* | Human | GGATAAGAAGCTAACCAAAGCCC | CTCCCAGTAAGAGATGTCCTGAT | 119 |
| *RNF144B* | Human | CATCTGGACCCCTACCGAACA | ACACGAGCAGAATTTCAGGTG | 129 |
| *ARPC5* | Human | TGGTGTGGATCTCCTAATGAAGT | CACGAACAATGGACCCTACTC | 128 |
| *UCN* | Human | CAACCCTTCTCTGTCCATTGAC | CGAGTCGAATATGATGCGGTTC | 115 |
| *CLIC3* | Human | CCTCAAGGGCGTACCTTTCAC | GTCGCTGTCATAGAGCAGGA | 112 |
| *GPR125* | Human | GCGTCATTACGGTCTTTGGAA | ACGGCAATTCAAGCGGAGG | 199 |
| *UCHL1* | Human | AGCTGAAGGGACAAGAAGTTAG | TTGTCATCTACCCGACATTGG | 265 |
| *THY1* | Human | CAGAAGGTGACCAGCCTAAC | TTGCTAGTGAAGGCGGATAAG | 233 |
| *RET* | Human | ATGAGAACAACTGGATCTGCAT | GAAGAAGGAGAAGTATACGCGG | 209 |
| *MAGEA4* | Human | CTTACCCACTACCATCAGCTTC | TGATGACTCTCTCCAGCATTTC | 212 |
| *PLZF* | Human | GGACAAGGTTGAGGAAAGAGG | CAACACGGAGTAGATGCCCAG | 205 |
| *SV40* | Human | GAACAGCCCAGCCACTATAA | ACTCCAGCCATCCATTCTTC | 248 |
| *YAP1-P1* | Mouse | GTCTTTCTCTAGGCACAAAAAGG | AGTGGTAAAGAATAATGCTCATCC | 400 |
| *YAP1-P2* | Mouse | GTCTTTCTCTAGGCACAAAAAGG | GTTTTGTTTTGTGCCAGGC | 1294 |
| *Stra8-Cre-P1* | Mouse | GATGGATTTCCGTCTCTGGTGTAG | CCCATTTAATCTCCTCCTTCTCCG | 443 |
| *Stra8-Cre-P1* | Mouse | GTCAGAGAAGGTTGTATCGAACTGG | CCCATTTAATCTCCTCCTTCTCCG | 264 |

**Table S5. The detailed information of antibodies for immunocytochemistry and immunohistochemistry**

| **Antibodies** | **Host species** | **Dilution** | **Companies** | **Catalog numbers** |
| --- | --- | --- | --- | --- |
| UCHL1 | Rabbit | 1:50 | CST | 13179 |
| UCHL1 | Mouse | 1:100 | Bio-rad | MCA4750GA |
| YAP1 | Rabbit | 1:100 | CST | 14074 |
| P-YAP1 | Rabbit | 1:100 | CST | 4911 |
| RAD21 | Rabbit | 1:1000 | Abcam | ab217678 |
| GFRA1 | Mouse | 1:100 | R＆D | MAB7141 |
| VASA | Goat | 1:20 | Santa | sc48705 |
| PLZF | Rabbit | 1:20 | Santa | sc22839 |
| GPR125 | Rabbit | 1:100 | Abcam | ab51705 |
| THY1 | Rabbit | 1:100 | Abcam | ab133350 |
| SV40 | Mouse | 1:50 | Santa | SC147 |
| Nuclei | Mouse | 1:20 | Millipore | MAB4383 |
| Alexa Fluor 555 | Donkey anti-Mouse | 1:1000 | Thermo scientific | A31570 |
| Alexa Fluor 488 | Donkey anti-Rabbit | 1:1000 | Thermo scientific | A21206 |
| Alexa Fluor 555 | Donkey anti-Rabbit | 1:1000 | Thermo scientific | A31572 |
| Alexa Fluor 488 | Donkey anti-Mouse | 1:1000 | Thermo scientific | A21202 |
| Alexa Fluor 555 | Donkey anti-Goat | 1:1000 | Thermo scientific | A21432 |

**Table S6. The siRNA sequences for PDK1, YAP1, NEDD4, and RAD21 oligonucleotides**

| **SiRNAs** | **Forward sequences (5'-3')** | **Reverse sequences (5'-3')** |
| --- | --- | --- |
| PDK1 siRNA1 | UUCCCUAAGGCAAGAGACCTT | GGUCUCUUGCCUUAGGGAAT |
| PDK1 siRNA3 | AAAUUCUUCCCUAAGGCAATT | UUGCCUUAGGGAAGAAUUUTT |
| YAP1 siRNA1 | GACGACCAAUAGCUCAGAUTT | AUCUGAGCUAUUGGUCGUCTT |
| YAP1 siRNA2 | GGUGAUACUAUCAACCAAATT | UUUGGUUGAUAGUAUCACCTT |
| YAP1 siRNA3 | CUGCCACCAAGCUAGAUAATT | UUAUCUAGCUUGGUGGCAGTT |
| NEDD4 siRNA1 | GCACUAGUGCUAAAGGAUUTT | AAUCCUUUAGCACUAGUGCTT |
| NEDD4 siRNA2 | GCAUCGAGCUCAAAUCAUUTT | AAUGAUUUGAGCUCGAUGCTT |
| NEDD4 siRNA3 | CCAAUGAUCUAGGGCCUUUTT | AAAGGCCCUAGAUCAUUGGTT |
| RAD21 siRNA1 | CCAAAGCCCAUGUGUUCGATT | UCGAACACAUGGGCUUUGGTT |
| RAD21 siRNA2 | CACUGCCUGACUUAGAUGATT | UCAUCUAAGUCAGGCAGUGTT |
| RAD21 siRNA3 | CAGAGCACCAGCAAUCUGATT | UCAGAUUGCUGGUGCUCUGTT |
| Control siRNA | UUCUCCGAACGUGUCACGUTT | ACGUGACACGUUCGGAGAATT |
| Cy3-labeled siRNA | UUCUCCGAACGUGUCACGUTT | ACGUGACACGUUCGGAGAATT |

**Table S7. The detailed information of antibodies for Western blots and Co-IP**

| **Antibodies** | **Host species** | **Dilutions** | **Companies** | **Catalog numbers** |
| --- | --- | --- | --- | --- |
| YAP1 | Rabbit | WB:1:1000 IP: 1:50 | CST | 14074 |
| YAP1 | Mouse | 1:1000 | CST | 12395 |
| P-YAP1 | Rabbit | 1:1000 | CST | 4911 |
| PDK1 | Rabbit | WB:1:2000 IP:1:50 | Abcam | ab207450 |
| PCNA | Mouse | 1:1000 | Abcam | ab29 |
| NEDD4 | Rabbit | WB:1:1000 IP: 1μg | Proteintech | 21698-1-AP |
| RNF144B | Rabbit | 1:1000 | Proteintech | 26306-1-AP |
| ARPC5 | Rabbit | 1:1000 | Proteintech | 16717-1-AP |
| RAD21 | Rabbit | 1:1000 | Abcam | ab217678 |
| RAD21 | Rabbit | WB:1:1000 IP:1:100 | Invitrogen | PA5-120663 |
| IgG | Mouse | IP: 1μg | Proteintech | B900620 |
| IgG | Rabbit | IP: 1μg | CST | 2729 |
| Histone-H3 | Rabbit | 1:8000 | Proteintech | 17168-1-AP |
| Tubulin | Mouse | 1:2000 | Abmart | M20005M |
| ACTB | Mouse | 1:1000 | CST | 3700 |
| HRP-IgG | Goat anti-Rabbit | 1:1000 | Beyotime | A0208 |
| HRP-IgG | Goat anti-Mouse | 1:1000 | Beyotime | A0216 |
